# Interactions of Brominated Flame Retardants with Membrane Models of Dehalogenating Bacteria: Langmuir Monolayer and Grazing Incidence X-ray Diffraction Studies

**DOI:** 10.1021/acs.langmuir.4c00518

**Published:** 2024-05-09

**Authors:** Marcin Broniatowski, Paweł Wydro

**Affiliations:** † Department of Environmental Chemistry, Faculty of Chemistry, the Jagiellonian University in Kraków, ul. Gronostajowa 2, Kraków 30-387, Poland; ‡ Department of Physical Chemistry and Electrochemistry, Faculty of Chemistry, the Jagiellonian University in Kraków, ul. Gronostajowa 2, Kraków 30-387, Poland

## Abstract

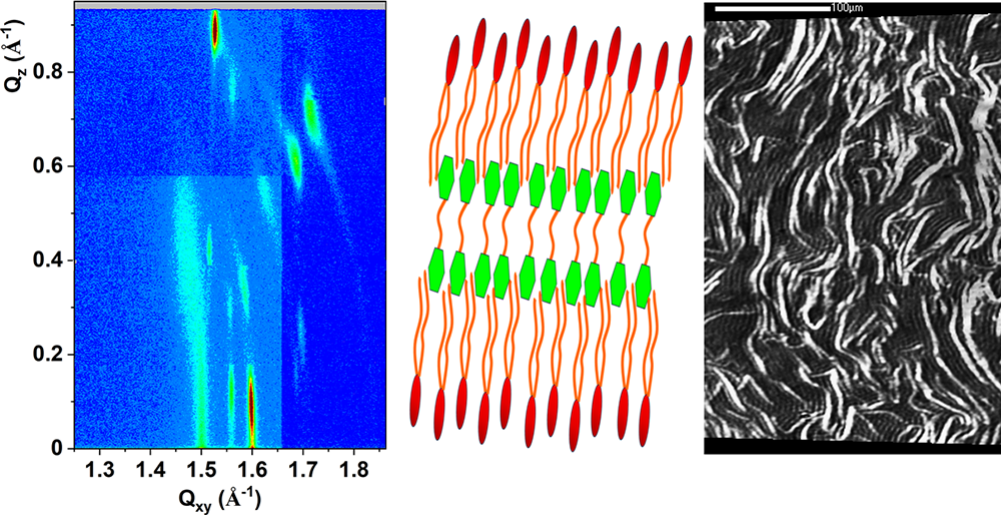

Brominated flame retardants (BFRs) are small organic molecules containing several bromine substituents added to plastics to limit their flammability. BFRs can constitute up to 30% of the weight of some plastics, which is why they are produced in large quantities. Along with plastic waste and microplastic particles, BFRs end up in the soil and can easily leach causing contamination. As polyhalogenated molecules, multiple BFRs were classified as persistent organic pollutants (POPs), meaning that their biodegradation in the soils is especially challenging. However, some anaerobic bacteria as *Dehaloccocoides* can dehalogenate BFRs, which is important in the bioremediation of contaminated soils. BFRs are hydrophobic, can accumulate in plasma membranes, and disturb their function. On the other hand, limited membrane accumulation is necessary for BFR dehalogenation. To study the BFR-membrane interaction, we created membrane models of soil dehalogenating bacteria and tested their interactions with seven legacy and novel BFRs most common in soils. Phospholipid Langmuir monolayers with appropriate composition were used as membrane models. These membranes were doped in the selected BFRs, and the incorporation of BFR molecules into the phospholipid matrix and also the effects of BFR presence on membrane physical properties and morphology were studied. It turned out that the seven BFRs differed significantly in their membrane affinity. For some, the incorporation was very limited, and others incorporated effectively and could affect membrane properties, while one of the tested molecules induced the formation of bilayer domains in the membranes. Thus, Langmuir monolayers can be effectively used for pretesting BFR membrane activity.

## Introduction

Plastic production has grown exponentially since the 1950s, reaching the level of 367 megatons in 2020.^1,2^ Plastics are blends of polymers and multiple nonpolymeric additives, giving them the required properties.^3^ Typical monomers for polymer production are petroleum-based substances; therefore, pure polymers are often highly flammable. To reduce the risk of fire, special substances called flame retardants are added to plastics, often constituting 20 to 30% of the weight of finished products.^4^ The most widespread on the market are brominated flame retardants (BFR) – small organic molecules containing several bromine substituents.^5,6^ In the beginning, in the 1960s and 1970s, polybrominated diphenyl ethers,^7^ polybrominated biphenyls,^8^ and tetrabromobisphenol A^9^ were applied as effective BFRs. Later, hexabromocyclododecane joined them on the market.^10^ The production of these substances in 2000, known now in the literature as legacy BFRs, exceeded 200000 tons.^6^ However, over time, it turned out that the legacy BFRs when released into the environment are toxic, persistent to biodegradation, and bioaccumulative, so they meet the definition of persistent organic pollutants (POPs).^11^ Therefore, polybrominated diphenyl ethers, polybrominated biphenyls, and hexabromocyclododecane have been included in Annex A of the Stockholm Convention, which means that their production and use are generally banned, with some exceptions allowed.^12,13^ The withdrawn BFRs have been replaced by new ones, possibly less persistent and bioaccumulative, as there is a constant demand for BFRs on the market.^14,15^ The introduction of new polybrominated molecules to the market led to the situation in which some BFRs are recognized as “legacy” and some as “novel”. This division is quite artificial, as some of the “novel” BFRs can be on the market for decades. Exposed to mechanical, physical, and chemical factors, plastic wastes fall apart forming microplastic particles, that is particles with dimensions smaller than 5 mm.^2,16^ Microplastics are emerging pollutants accumulating mainly in soils and bottom sediments.^17−19^ Most BFRs and other plastic additives are not covalently bonded with polymer macromolecules and can relatively easily leach into the soil leading to contamination.^3,20^ The persistence of BFRs and many other POPs originates from the substitution of an organic molecule with multiple halogen atoms. Polyhalogenated organics accumulated in the soil are often toxic to soil microorganisms and the contamination can lead to the depletion of soil microflora, lowering the rate of organic matter degradation, and ultimately the reduction of soil fertility.^21,22^ Fortunately, some soil bacteria produce dehalogenases, that is, enzymes that cleave halogen atoms.^23^ Especially important here is the process of halorespiration – for some bacteria, halogenoorganic (chlorinated or brominated) molecules are essential to life being the terminal electron acceptors in their anaerobic respiration process.^24^ Among them, *Dehalococcoides* is the most widespread and the best-researched genus.^25,26^ These small Gram-positive bacteria are widespread in soils, sediments, and porous aquifer rocks. They can subsequently dehalogenate even perhalogenated aromatic organics, such as hexachlorobenzene,^27^ and different highly brominated diphenyl ethers.^28,29^ To perform dehalorespiration, *Dehalococcoides* need gaseous hydrogen, which they cannot produce themselves, thus cooperation in consortium with other bacteria is crucial for their effectiveness.^25−28^*Rhodopseudomonas palustris* is a Gram-negative versatile photosynthetic bacterium that can also degrade BFRs, but what is more, emits hydrogen.^30,31^*Dehalococcoides* and *Rhodopseudomonas palustris* are frequently applied in bioremediation processes, both in the *ex situ* and also *in situ* techniques.^32−35^ They could also be effectively used to remediate BFR-contaminated soils.^36−38^ However, BFRs, and their metabolites, are often toxic to different members of soil microbial consortia.^21,22^ BFRs are hydrophobic and when absorbed from the soils they may damage the plasma membranes of the bacterial cells.^38−40^ To be effectively metabolized, BFRs must be incorporated into the membrane, as multiple enzymes, including dehalogenases, are membrane-related.^41,42^ Accumulated in the membrane, BFRs should not disturb its structure and impair functions.

Bacterial plasma membranes are structures with a high degree of complexity; thus, in science reductionist models are frequently applied.^43^ It was also our idea to construct simplified phospholipid models of *Dehalococcoides* and *Rhodopseudomonas palustris* membranes and apply them in studies on the impact of selected BFRs on model bacterial membranes. As model membranes, we applied Langmuir monolayers^44^ formed of phospholipids typical to soil bacteria having dehalogenating activity. Now, in the era of the circular economy, all stages of the product life cycle should be taken into account. Regardless of the efforts made, some part of the produced plastic will always turn into microplastic and end up in the soil.^2,17^ Therefore, modern plastics should use BFRs that are not toxic to soil bacteria, thanks to which their fast biodegradation will prevent soil contamination. The membrane activity of a given BFR can be quickly prescreened on membrane models, providing information about its possible toxicity and the perspectives of its biodegradation. In our studies, we used both the legacy BFRs, which due to their persistence are still detected in polluted soils, and also representatives of novel BFRs, trying to use in the studies BFRs with the highest production tonnage.^45^

## Experimental Section

### Materials

All phospholipids used in the studies are the following: 1,2-dioleoyl-*sn*-glycero-3-phosphocholine (DOPC), 1,2-dipalmitoyl-*sn*-glycero-3-phosphocholine (DPPC), 1,2-dioleoyl-*sn*-glycero-3-phosphoethanolamine (DOPE), 1-palmitoyl-2-oleoyl-*sn*-glycero-3-phosphoethanolamine (POPE), 1,2-dipalmitoyl-*sn*-glycero-3-phosphoethanolamine (DPPE), 1,2-dioleoyl-*sn*-glycero-3-phospho-(1′-rac-glycerol) sodium salt (DOPG), and 1,2-dipalmitoyl-*sn*-glycero-3-phospho-(1′-rac-glycerol) sodium salt (DPPG) were purchased from Avanti Polar Lipids. All the phospholipids were powdered, lyophilized samples, of 99% purity. BFRs used in the studies: 3,3′,5,5′-tetrabromobisphenol A (TBBPA), 2,2′,4,4′,5-pentabromodiphenyl ether (BDE 99), 1,2,5,6,9,10-hexabromocyclododecane (HBCD), 1,2,5,6-tetrabromocyclooctane (TBCO), 1,2-bis(2,4,6-tribromophenoxy)ethane (BTBPE), 2,4,6-tribromophenol (TBP), and 2,3,4,5,6-pentabromotoluene (PBT) were purchased from Merck Signa-Aldrich. All the BFRs were analytical standards of purity >99%. The applied organic solvents: chloroform (99.5%, HPLC grade) and methanol (99.5%, HPLC grade) were purchased from Merck Sigma-Aldrich. Ultrapure water of 18.2 MΩ·cm resistivity was produced in our laboratory with the application of a Merck MilliPore water purification system.

### BFRs’ Selection

We intended to use in the research both legacy and novel BFRs. The criteria for their selection were their production tonnage and their presence in polluted soils proven by scientific literature. TBBPA is the BFR produced in the largest quantity exceeding 200 kilotons yearly.^9^ It is a reactive BFR applied mainly in bisphenol A-related plastics, like polycarbonates and epoxy resins. Due to covalent bonding to the polymer, its leaching from MPs is limited; however, due to its large tonnage of production, TBBPA is an emerging soil contaminant. BDE 99 is a representative of the banned PBDEs. There are 209 congeners of PBDEs^7^; however, only some of them are formed during the bromination of diphenyl ether and therefore are present in industrial formulations and can be detected in the environment. BDE 99 is the most widespread congener of pentabrominated congeners, and taking under consideration its concentration in soils, of all PBDEs in the environment.^6^ HBCD is the last legacy BFR in the studies, also banned by the Stockholm Convention, but due to its wide production in the previous decades and its leaching from MPs, is still present in contaminated soils.^10^ TBCO is, like HBCD, a polybrominated cycloalkane, but its use as a plastic additive is not legally restricted. However, TBCO is also accused of being ecotoxic.^46^ BTBPE is one of the most commonly used novel BFRs,^45^ applied especially in all these plastics, where PBDEs were previously used. Similar to HBCD and TBCO, BTBPE exhibits cytotoxicity and can damage plasma membranes.^47^ TBP is produced in quantities of 10 to 100 tons yearly and applied as novel BFR in some plastics.^45^ Moreover, TBP is a BFR of special environmental concern, as it is also a degradation product of multiple other BFRs, to name only PBDEs or BTBPE.^48^ Finally, PBT is also a novel BFR often detected in environmental matrices.^45^ Like TBP, it is a small molecule with a polybrominated benzene ring, but it differs from TBP in much greater hydrophobicity.

**Scheme 1:**
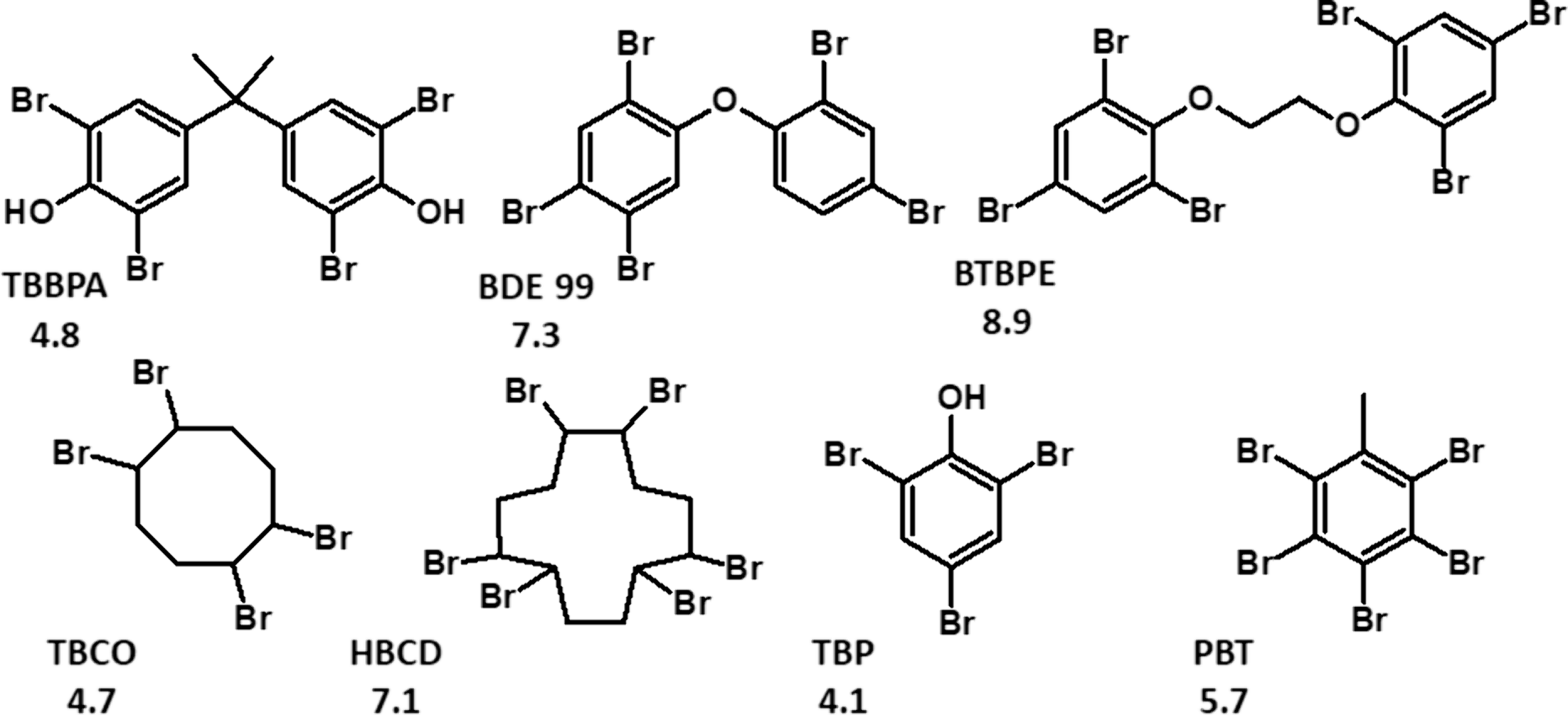
Structural Formulae of the Investigated BFRs The numbers below the acronyms are the values of p*K*_OW_ of these BFRs.^49,50^

The structural formulas of the investigated BFRs are illustrated in Scheme 1.

### Modeling the Membranes of Dehalogenating Bacteria

*Rhodopseudomonas palustris* is a Gram-negative bacterium with a rather unusual composition of the inner plasma membrane. Unlike most prokaryotes, it contains in its inner plasma membrane significant amounts of phosphatidylcholines (PCs). Following the literature data, the proportion of the main phospholipid classes are 50% phosphatidylethanolamines (PEs), 40% phosphatidylcholines, and 10% phosphatidylglycerols (PGs)/cardiolipins (mole percent of all membrane phospholipids).^51−53^ We adopted this proportion for the construction of our models. Taking into account the data on the distribution of fatty acid chains,^52^ and the fact that monounsaturated fatty acids dominate, we decided that in the realities of our research, 1,2-dioleoyl-phospholipids, that is DOPE, DOPC, and DOPG, would be the best choice. Thus, the first model MRU (u from unsaturated) mimics the inner plasma membranes of *R. palustris* in favorable environmental conditions. As for *Dehalococcoides*, to the best of our knowledge, there are no detailed data regarding the composition of its plasma membrane. *Dehalococcoides* are Gram-positive bacteria; thus, following the literature data,^54,55^ negatively charged phospholipids, that is PGs and cardiolipins (CLs), dominate in their membranes, containing up to 80% of all phospholipids, whereas the remaining 20% are PEs. Based on our previous studies regarding the modeling of plasma membranes of soil bacteria^56^ and to be consistent with the model proposed for *R. palustris*, we constructed a model membrane MDU consisting of 80% DOPG and 20% DOPE. Exposed to unfavorable environmental conditions or hydrophobic toxicants, bacteria adjust the composition of their membranes by replacing unsaturated fatty acid chains with saturated.^57−59^ Therefore, we constructed and studied also membrane models composed of saturated phospholipids: MRS composed of 50% DPPE, 40% DPPC, and 10% DPPG mimicking the inner membrane of *R. palustris*, and MDS composed of 80% DPPG and 20% DPPE mimicking the membrane of *Dehalococcoides*. It should be underlined that in nature, a plasma membrane cannot contain solely saturated phospholipids. Thus, of course the MRS and MDS models simplify the reality of the membrane; however, the simplified models can still provide a more complete understanding. The compositions of the models are summarized below.

MRU: 50% DOPE, 40% DOPC, 10% DOPG.

MDU: 80% DOPG, 20% DOPE.

MRS: 50% DPPE, 40% DPPC, 10% DPPG.

MDS: 80% DPPG, 20% DPPE.

### Solution Preparation and Langmuir Monolayer Technique

The BFRs and phospholipid samples were weighed on a Mettler Toledo analytical scale with an accuracy of 10 μg. The samples were dissolved in 10 mL volumetric flasks in a chloroform/methanol 9/1 v/v mixture. The concentrations of the BFR solutions ranged from 0.1 to 0.15 mg/mL, whereas for the phospholipids, it was 0.2 to 0.3 mg/mL. The stock solutions were stored at −20 °C. The mixtures MRU, MDU, MRS, and MDS were prepared in 5 mL volumetric flasks by mixing appropriate volumes of the phospholipid stock solutions. Mixtures with the addition of a given BFR were prepared in glass vials just before an experiment.

Three Langmuir troughs were used in our studies. π–*A* isotherms were measured on a KSV NIMA double-barrier trough of the area of 273 cm^2^. Brewster angle microscopy experiments were performed on a larger KSV NIMA Langmuir trough of the area 580 cm^2^ designed by the manufacturer to work with this microscope. A custom-made single-barrier R&K Langmuir trough of the area of 500 cm^2^ is installed in the Sirius beamline of the SOLEIL synchrotron. Ultrapure Milli-Q water was applied as a subphase. We did not use buffers so as not to complicate the tested system and to be able to attribute the observed changes in isotherms or monolayer texture directly to BFR-phospholipid interactions and not to buffer components-phospholipid interactions. Appropriate volumes of the chloroform solutions were deposited with Hamilton analytical syringes at the air/water interface. Ten minutes were left for solvent evaporation, and then the monolayers were compressed with a compression rate of 20 cm^2^·min^–1^. Surface pressure (π) was measured with a Wilhelmy-type electrobalance (KSV NIMA) with a rectangular plate of filtration paper (Whatmann, ashless) used as a surface pressure sensor. The accuracy of π measurements was 0.05 mN/m. Each experiment (π–*A* isotherm recording) was repeated at least 3 times, and the uncertainty of the estimation of mean molecular area (A) was 1 Å^2^/molecule. All experiments were performed at 20 ± 0.1 °C, and the subphase temperature was controlled by a water-circulating bath (Julabo). Compression modulus C_S_^–1^ was calculated from the course of the π–*A* isotherms, according to its definition:^60^
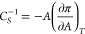


None of the studied BFRs is surface active nor forms Langmuir monolayers. The seven studied BFRs can also be treated as completely water-insoluble in the applied experimental conditions. When added to the phospholipid solution the BFR molecules can either incorporate into the phospholipid monolayer, form 3D aggregates on the monolayer/air interface, or form an adsorptive layer on top of the monolayer. Therefore, in all the experiments, the number of phospholipid molecules deposited at the air/water interface was constant, so the mean molecular area *A* is the mean molecular area per phospholipid molecule.

### Brewster Angle Microscopy

A Brewster angle microscopy UltraBAM instrument (Accurion GmbH, Goettingen, Germany) equipped with a 50 mW laser emitting p-polarized light at a wavelength of 658 nm, a 10× magnification objective, polarizer, analyzer, and a CCD camera was used. The spatial resolution of the microscope was 2 μm. The foregoing apparatus and the Langmuir trough were placed on a table (Standa Ltd., Vilnius, Lithuania) equipped with an active vibration isolation system (antivibration system VarioBasic 40, Halcyonics, Göttingen, Germany).

### Grazing Incidence X-ray Diffraction

GIXD experiments were performed on the Sirius beamline of the SOLEIL synchrotron (Gif sur Yvette, France) using a dedicated surface diffractometer.^61,62^ The description of the settings of this instrument and the performance of a routine experiment can be found in the experimental section of ref (63).

## Results and Discussion

### Characterization of the Model Membranes of Halorespiring Bacteria

**Figure 1:**
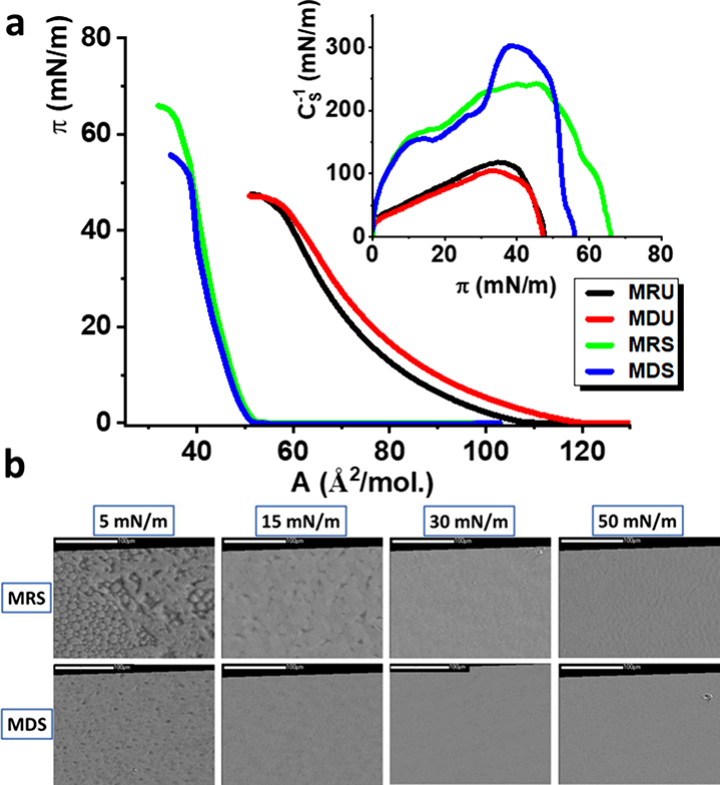
(a) π–*A* isotherms and C_S_^–1^–π curves for the monolayers mimicking the membranes of halorespiring bacteria and (b) selected BAM images for the models MRS and MDS. The scale bar is 100 μm.

At the beginning of our research, one-component Langmuir monolayers from all six phospholipids used for the formation of the membrane models were prepared and studied. The resultant π–*A* isotherms and C_S_^–1^–π curves are presented in Figure S1 of the Supporting Materials. Generally, all the curves were consistent with the literature data. Then, the multicomponent monolayers MRU, MDU, MRS, and MDS were prepared and studied. The π–*A* isotherms, C_S_^–1^–π curves, and selected BAM images for these systems are presented in Figure 1.

The MRU and MDU models are formed from dioleoyl phospholipids, which typically form Langmuir monolayers in the liquid expanded state (LE).^64,65^ Monolayers in this state are easily compressible, which results in low values of compression modulus, usually not exceeding 100 mN/m.^60^ The lift-off area, *A*_0_ for MRU is 108 Å^2^/mol. During compression, surface pressure increases slowly, and the monolayer collapses at the π = 46 mN/m and the area of 57 Å^2^/mol. The isotherm for the MDU monolayer has a similar course but is shifted toward greater mean molecular areas, *A*_0_ = 119 Å^2^/mol. The charged molecules of DOPG dominate in this model; thus, the repulsion between the negatively charged headgroups increases the distance between the film-forming molecules, resulting in the shift of the isotherm and slightly lower values of compression modulus at higher π values. The isotherms measured for the saturated MRS and MDS models are shifted to much lower mean molecular areas – *A*_0_ of 52 Å^2^/mol. The isotherms are very steep and practically overlap, differing only in the collapse parameters (π = 64 mN/m and *A* = 36 Å^2^/mol for MRS and π = 52 mN/m and *A* = 38 Å^2^/mol for MDS). For both the monolayers, C_S_^–1^ achieves the value of 100 mN/m at π below 5 mN/m, so according to the Davies and Rideal criterion,^60^ they are mainly in the liquid condensed (LC) state. For MDS for high π values, C_S_^–1^ exceeds even the threshold value of 250 mN/m, so the ordering of the hydrocarbon chains typical to the solid (S) state of a monolayer is here also possible. All the model membranes were visualized upon their compression with the application of Brewster angle microscopy. The liquid-expanded MRU and MDU monolayers were dark and homogeneous in the BAM images from the beginning of the compression until the collapse of the monolayers. No condensed domains or 3D aggregates were observed for them. At the compression of the MRS and MDS monolayers, multiple condensed domains were observed already at very low surface pressure values (Figure 1b). At 5 mN/m, these domains are partially coalesced (MRS) and almost fully coalesced with residual holes (MDS). For MRS at 15 mN/m, these domains are partially fused but some boundaries between them are still visible, whereas the monolayer of MDS is practically homogeneous under those conditions. At 30 mN/m, both MRS and MDS monolayers are completely homogeneous and remain homogeneous at 50 mN/m. The lack of 3D aggregates at 50 mN/m confirms the stability of these model membranes.

**Figure 2:**
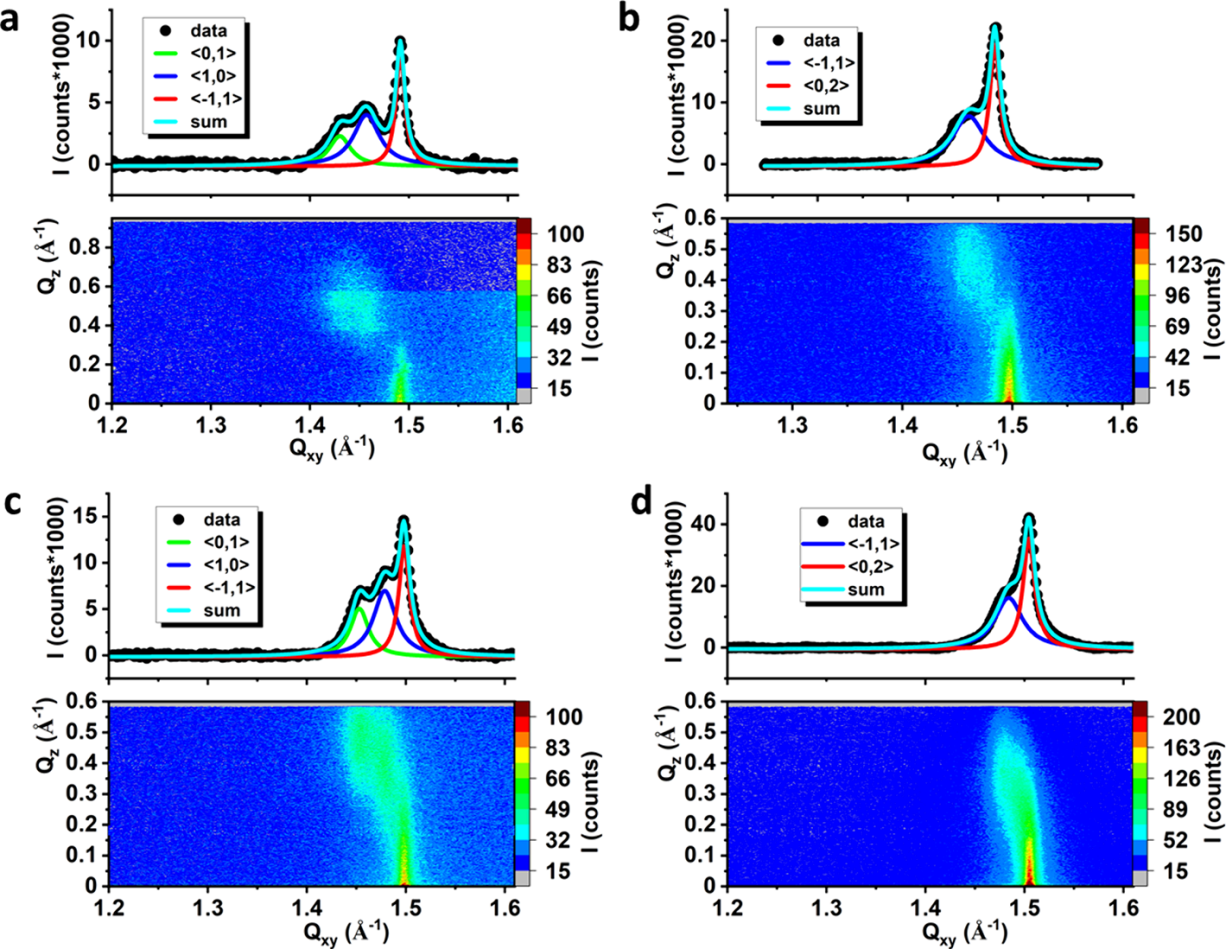
GIXD results: I(Q_*xy*_,Q_*z*_) intensity maps and I(Q_*xy*_) Bragg peak profiles integrated over all Q_*z*_ values for the saturated models of dehalorespiring bacteria membranes: (a) MRS π = 10 mN/m, (b) MRS π = 20 mN/m, (c) MDS π = 10 mN/m, (d) MDS π = 20 mN/m. Solid lines in Bragg peak profiles are Lorentz curves fitted to the experimental data.

The components of the MRS and MDS model membranes, that is DPPC, DPPE, and DPPG, form 2D crystalline Langmuir monolayers when spread at the air/water interface^66−68^; thus, we employed the GIXD technique to study the MRS and MDS membrane models. The GIXD experiments for both model membranes were performed at π = 10 and 20 mN/m, as these values correspond to further studies performed on the BFR-doped monolayers. Frequently in the literature 30 mN/m is accepted as a surface pressure at which the packing of hydrocarbon chains in a Langmuir monolayer corresponds to the packing in a bilayer.^44^ However, as will be discussed later, the BFR molecules separate from the model membranes upon their compression; therefore, the possible inclusion of BFR molecules into the 2D crystalline phases was studied at 10 and 20 mN/m. The results are presented in Figure 2, whereas the structural parameters extracted from the GIXD data are summarized in Table 1.

**Table 1:** Structural Parameters Extracted from the GIXD Data^a^

**model**	**Q**_*xy*_**, Q**_*z*_**(Å**^**–1**^**, Å**^**–1**^**)**	**a, b, γ (Å, Å, deg)**	**A**_*xy*_**(Å**^**2**^**)**	**τ (deg)**	**L**_*xy*_**(Å)**	**I (a.u.)**
MRS, π = 10 mN/m	**⟨0,1⟩** 1.433; 0.58 **⟨1,0⟩** 1.464; 0.49 **⟨-1,1⟩** 1.498; 0.02	4.849; 4.954; 117.7	21.26	23.4	197 ± 21 162 ± 9 595 ± 32	363
MRS, π = 20 mN/m	**⟨-1,1>** 1.452; 0.46 **⟨0,2⟩** 1.484; 0	4.979; 8.400; 90	20.91	20.0	126 ± 3 395 ± 8	860
MDS, π = 10 mN/m	**⟨0,1⟩** 1.453; 0.50 **1,0⟩** 1.479; 0.38 **⟨-1,1**⟩**** 1.498; 0.02	4.837; 4.923; 118.6	20.92	19.7	251 ± 9 197 ± 7 425 ± 10	658
MDS, π = 20 mN/m	**⟨-1,1**⟩**** 1.484; 0.33 **⟨0,2**⟩**** 1.504; 0	4.911; 8.355; 90	20.51	14.5	163 ± 4 395 ± 7	1434

a Q_*xy*_, Q_*z*_ – components of the scattering vector, a, b, γ – 2D lattice parameters, A_*xy*_ – area per hydrocarbon chain, τ – tilt angle of the hydrocarbon chain from the monolayer normal, L_*xy*_ range of 2D crystallinity (following the Scherrer formula L_*xy*_ = 0.9*2π/fwhm_Bragg peak_, where fwhm is the full width at half-maximum^69,70^), I – integrated intensity of the diffraction signal.

The GIXD measurements confirmed that 2D crystalline nanodomains form both in MRS and MDS, at low surface pressures, as a diffraction signal, quite intense for MDS, can be observed already at π = 10 mN/m. For MRS at π = 10 mN/m, the diffraction signal is triply degenerated and the sequence of the diffraction maxima is typical of the oblique 2D lattice. At π = 20 mN/m, the ⟨0,1⟩ and ⟨1,0⟩ maxima merge, and the Bragg peak profile is best fitted with two Lorentz curves. The intensity ratio of both signals and their location in the Q_*xy*_, Q_*z*_ plane prove that the rectangular-centered lattice best describes the ordering of the hydrocarbon chains. For MDS also, three diffraction peaks are observed at π = 10 mN/m, identifying the 2D oblique lattice. At 20 mN/m, all the maxima approach each other, indicating a significant lowering of the tilt of the hydrocarbon chains. Their packing within the monolayer plane can be described by the rectangular-centered lattice. The greater tilt angle observed for the MRS monolayer originates from the presence of DPPC in this model. DPPC has a larger headgroup than DPPE and DPPG, which induces a greater tilt of the hydrocarbon chains.^67^ Regarding the lattice type, an oblique lattice was proposed for both models at 10 mN/m (three peaks fitted), while at 20 mN/m, a rectangular centered lattice was more probable (two peaks fitted). The shift from the oblique to the rectangular centered lattice with increasing surface pressure can originate from the ordering effect of DPPE, present in both models. One-component DPPC and DPPG monolayers are described by the oblique lattice even at high π values,^66,68^ whereas for DPPE, the rectangular centered lattice was proposed even at very low surface pressure values (2 mN/m).^69,70^

### Interactions of BFRs with the Model Membranes MRU and MDU

In our studies, we tested 7 BFRs. Looking at Scheme 1, some structural similarities between these compounds can be noticed. TBBPA, BDE 99, and BTBPE have two polybrominated benzene rings in their molecules. HBCD and TBCO are polybrominated cycloalkanes, while TBP and PBT are derivatives of polybrominated benzene. Thus, in further figures, these compounds will be grouped. The monolayers were doped with BFRs so that the molar ratio of these impurities was 0.1 or 0.2. We begin the presentation of the results for the unsaturated model of the *R. pseudomonas* membrane, MRU. The π–*A* isotherms and C_S_^–1^–π curves for the MRU monolayers doped in the studied BFRs are presented in Figures S2, S3, and S4 in the Supporting Materials. In the main manuscript, we present the zoomed sections of the isotherms for π ranging from 20 to 30 mN/m. As can be noticed in Figures S2 to S4, the isotherms measured for the BFR-doped monolayers are located close to each other, partially overlapping. The MRU monolayers are in the LE state, surface pressure grows slowly upon the monolayer compression, and no indicators of phase transitions (plateaus, kinks) are observed in their courses. Therefore, the zooming of a section of the isotherms facilitates the discussion of the effects of BFRs on the model membranes. The sections between π = 20 and 30 mN/m were selected, as 30 mN/m is a surface pressure at which the packing of the hydrocarbon chains most closely resembles the packing in a bilayer.^44^

For TBBPA, BDE 99, and BTBPE at X(BFR) = 0.1, all three isotherms for the doped monolayers are shifted 4–5 Å^2^/mol toward greater *A* values as compared with the isotherm recorded for the undoped MRU membrane. Taking under consideration that the addition of these BFRs either lowers the maximum value of C_S_^–1^ (BDE 99) or leaves it practically unchanged (TBBPA and BTBPE), the shift of the isotherms toward greater *A* values originates from the incorporation of the BFR molecules into the model membranes. For BDE 99, the isotherm measured at X(BDE 99) = 0.2 practically overlaps with the curve recorded at X(BDE 99) = 0.1. This means that the incorporation of this BFR into the MRU membrane is concentration-limited, that is, at X(BDE 99) = 0.2, some of the BDE 99 molecules are not built into the phospholipid membrane. For BTBPE, the shift between the curves measured at X(BTBPE) = 0.1 and 0.2 is 1.5 Å^2^/mol toward greater *A* values. Thus, changing the BTBPE mole ratio from 0.1 to 0.2 induces some further incorporation of BTBPE molecules into the membrane. However, the shift of only 1.5 Å^2^/mol between the curves is small; thus, probably part of the BTBPE molecules is separated from the MRU membrane. For TBBPA, the distance between the curves measured at X = 0.1 and 0.2 is the greatest (2 Å^2^/mol), so an initial conclusion can be drawn that from the BFRs having two benzene rings in their molecules, TBBPA incorporates most effectively to the model membrane. The doping of the MRU membrane with polybrominated cycloalkanes TBCO and HBCD also leads to the shifts of the π–*A* isotherm toward greater *A* values. For TBCO, this shift is 6 Å^2^/mol both for X(TBCO) = 0.1 and 0.2, as both the isotherms overlap ideally. It means that a limited number of TBCO molecules can be incorporated into the MRU membrane, and increasing the ratio from 0.1 to 0.2 does not lead to the incorporation of additional TBCO molecules. The situation is different for HBCD as the isotherm measured at X(HBCD) = 0.2 is shifted 6 Å^2^/mol toward greater *A* values than the curve measured at X = 0.1. Moreover, the increase in the HBCD mole ratio to 0.2 leads to a noticeable decrease in compression modulus. It means that HBCD compared to the other studied here BFRs exhibits an increased affinity toward phospholipid membranes and when incorporated lowers the organization degree of the phospholipid hydrocarbon chains. For MRU monolayers doped in TBP and PBT, all the π–*A* isotherms are shifted 6 Å^2^/mol toward greater *A* values. The curves recorded at X(BFR) = 0.1 and 0.2 overlap within the experimental error. This means that the incorporation of these molecules into the model membrane is limited and that the increase of the mole ratio of TBP or PBT from 0.1 to 0.2 does not lead to the incorporation of additional molecules into the model membrane.

All the MRU monolayers doped in the studied BFRs were visualized by Brewster angle microscopy, and the selected BAM images are presented in Figure S5 in the Supporting Materials. As already mentioned, MRU monolayers formed from unsaturated dioleoyl phospholipids remain in the LE state at all surface pressures until the monolayer collapse and are completely dark and homogeneous in BAM images. On the other hand, 3D aggregates are visible in BAM as bright white spots. Thus, these measurements were performed to identify the mole ratio of the added BFR and the value of surface pressure at which such aggregates occur. BAM images presented in Figure S5 were taken at π = 10, 20, and 30 mN/m. For the monolayer doped in TBBPA, virtually no aggregates were observed at X = 0.1, which confirms the entire incorporation of these BFR molecules into the model membrane. At X = 0.2, few aggregates were already visible at 10 mN/m, and as π increased to 20 mN/m, their number grew rapidly and they dominated the BAM images. The microscopic observations confirmed the conclusions drawn from the courses of the π–*A* isotherms. For BDE 99 at X = 0.1, no aggregates were visible at π = 10 and 20 mN/m, whereas at 30 mN/m, multiple 3D aggregates were observed. This means that upon the MRU membrane compression, the originally incorporated BDE 99 molecules are successively eliminated from the monolayer. At X = 0.2, numerous aggregates were observed already at low π values, proving the limited incorporation of this BFR to the model membrane. Regarding the polybrominated cycloalkanes TBCO and HBCD, no 3D aggregates were observed at X = 0.1. Meanwhile at X = 0.2, for the TBCO-doped monolayer, the aggregates appeared at π = 20 mN/m, whereas for HBCD some tiny aggregates can be discerned in the images only at 30 mN/m. This agrees with the conclusions drawn from measurements of π–*A* isotherms and confirms the increased membrane activity of HBCD. As for the polybrominated benzene derivatives, for TBP, no aggregates were seen at X(TBP) = 0.1, whereas at X = 0.2, they were observed at low surface pressure values. For PBT, multiple aggregates were observed already at X = 0.1 and low π values, which indicates that the incorporation of this BFR is very limited. We left BTBPE at the end of the discussion, as the BAM images for this compound were completely different from those discussed for the other six investigated BFRs. At X = 0.1 and π = 10 mN/m, long (some of them longer than 100 μm) filamentous structures were observed in BAM images. They were so bright that they had to be multilayered. At higher π values, small round 3D aggregates dominated in the view field of the microscope. However, at X = 0.2, only the filamentous structures were visible from very low surface pressures until the monolayer collapse. Practically identical filamentous structures were observed also for the saturated models MRS and MDS of the bacterial membranes (see Figure 5). Their possible origin and significance will be discussed later. It must be underlined that BTBPE alone when spread from chloroform solution at the air/water interface does not spread to a monolayer coverage but forms large multilayer islands presented in Figure S6 in the Supporting Materials. Formation of such islets (multilayer lenses) is typical when hydrophobic, not surface-active substances are spread at the water surface from a solution in a volatile organic solvent. BTBPE alone does not form any filamentous structures, so these structures result from the BTBPE-phospholipid interactions and should be formed from both BTBPE and phospholipid molecules.

**Figure 3:**
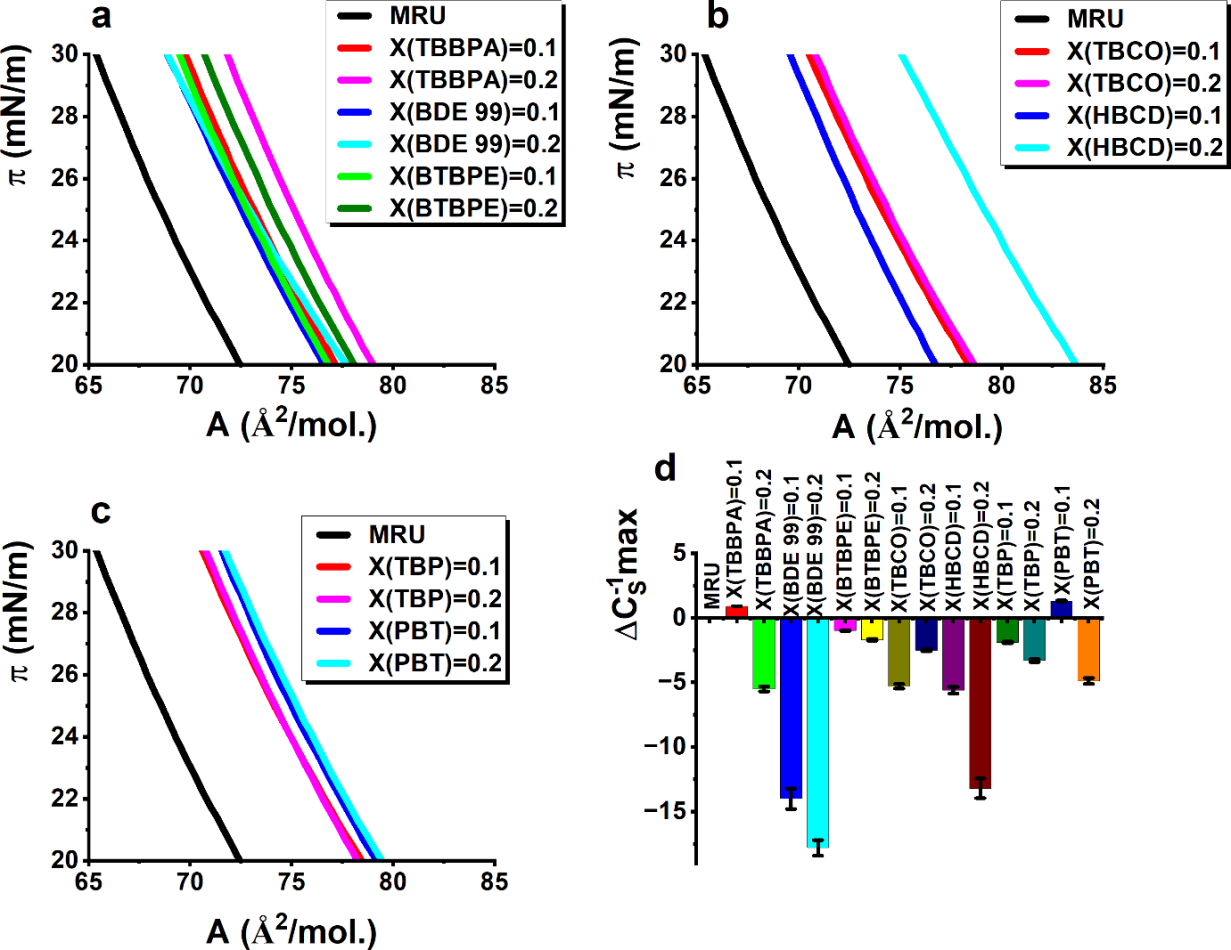
(a–c) π–*A* isotherms (sections from π = 20 to 30 mN/m) for the MRU monolayers doped in (a) TBBPA, BDE 99, BTBPE, (b) TBCO and HBCD, (c) TBP and PBT. (d) Difference between the maximal C_S_^–1^ values observed for a doped monolayer and the MRU model membrane.

An identical set of experiments was performed for the MDU model membranes. It turned out that the obtained results were qualitatively very similar to those obtained for the MRU model. A figure identically organized as Figure 3 was prepared; however, we decided to place it in the Supporting Materials (Figure S7), as the description of the effects of each BFR on the MDU would be in large a repetition of the above section. In the Supporting Materials, the reader can find also the plots of the π–*A* isotherms and C_S_^–1^, grouped in three figures – Figures S8, S9, and S10, following the division of the studied BFRs. The BFR-doped MDU membranes were also visualized by BAM to check the conditions at which 3D aggregates form. The results were practically identical to those presented in Figure S5, so they were not included in the Supporting Materials.

### Interactions of BFRs with the Model Membranes MRS and MDS

**Figure 4:**
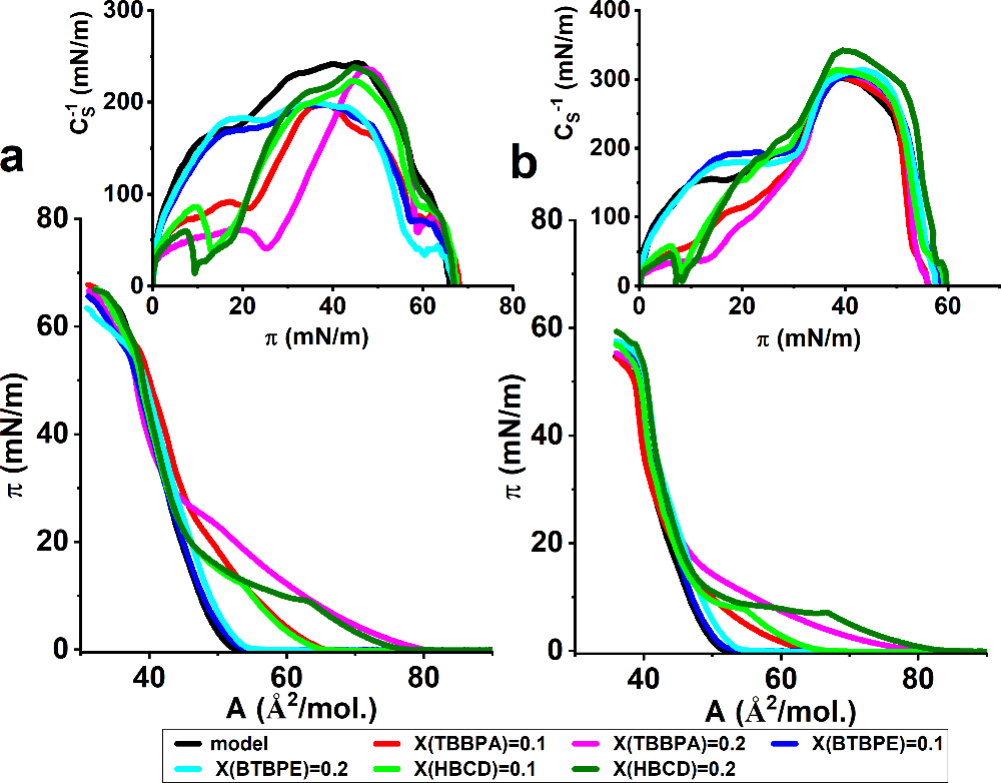
π–*A* isotherms and C_S_^–1^–π curves for BFR-doped model membranes (a) MRS and (b) MDS.

The model membranes prepared from saturated phospholipid, that is, MRS and MDS were also doped in the seven studied BFRs following the procedures described in the previous sections. It turned out that the introduction of TBBPA and HBCD led to significant expansion of the model membranes, especially at lower surface pressures. Moreover, for BTBPE, again the formation of filamentous domains was observed in the BAM images. For the other four BFRs, BDE 99, TBCO, TBP, and PBT, the changes in the course of the π–*A* isotherms and the C_S_^–1^–π curves were minor, and even less pronounced than for the unsaturated models. Thus, in the main manuscript, we included only the π–*A* isotherms and C_S_^–1^–π curves for the monolayers doped in TBBPA, HBCD, and BTBPE (Figure 4). The isotherms and compression moduli for the other four BFRs are presented in Figures S11 and S12 in the Supporting Materials. Moreover, as the effects of the incorporation of TBBPA, HBCD, and BTBPE were much more significant than for the other four BFRs, the BAM and GIXD studies were performed only for these three compounds.

For BTBPE-doped membranes at both X = 0.1 and 0.2, π–*A* isotherms overlap with the curves recorded for the undoped MRS and MDS membranes. Such a situation indicates that BTBPE molecules do not incorporate into the model membranes formed from saturated phospholipids. On the other hand, the addition of BTBPE lowers the values of C_S_^–1^, especially for the MRS model, thus affecting the elasticity of the monolayer. The changes in elasticity can originate from the formation of the multilayer filamentous structures, which will be discussed later in this section. The presence of TBBPA and HBCD significantly affects the properties of both model membranes, which manifests in the changes in π–*A* isotherm courses. In the presence of these BFRs, *A*_0_ for MRS increases from 52 Å^2^/mol to 65 Å/mol at X(BFR) = 0.1 and to 78 Å^2^/mol for X(BFR) = 0.2, while for MDS it is 63 Å^2^/mol at X = 0.1 (for both BFRs), 75 Å^2^/mol at X(TBBPA) = 0.2 and 82 Å^2^/mol at X(HBCD) = 0.2. The shift of the π–*A* isotherms toward greater mean molecular areas originates from the expansion of the monolayers manifesting in the significant decrease of C_S_^–1^ values. For MRS, the limiting value of 100 mN/m (the conventional boundary between the LE and LC states^60^) is achieved between π = 20 to 25 mN/m, and even at 32 mN/m for TBBPE at X = 0.2. For MDS, these trends are analogues but the value 100 mN/m in the C_S_^–1^–π plot is achieved at lower π values of 15 and 23 mN/m, respectively. It should be underlined that in the course of the π–*A* isotherms of the BFR-doped monolayers, a plateau region appeared, which manifests in the C_S_^–1^–π curves as a deep minimum. This plateau region in the isotherms appears due to a phase transition in the studied monolayer. This can be just the LE-LC transition, or the long plateau can originate from the gradual elimination of the originally incorporated BFR molecules from the monolayer induced by the rise in surface pressure. The second interpretation can be valid as from ca. 30 mN/m for MRS and ca. 20 mN/m for MDS, the isotherms for the BFR-doped and undoped monolayers approach each other and even overlap within the experimental error.

**Figure 5:**
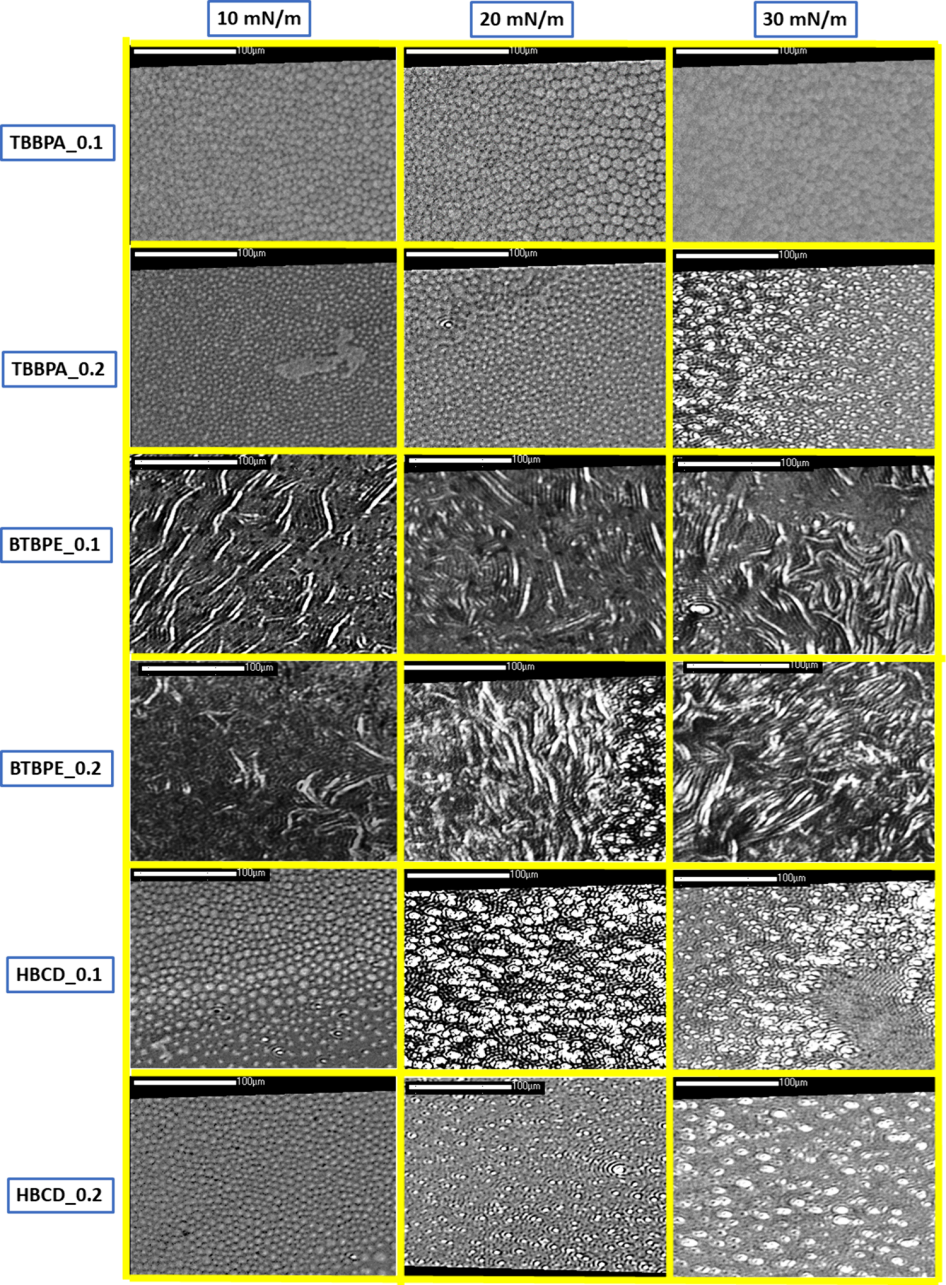
Representative BAM images for the MRS model membrane doped in TBBPA, BTBPE, and HBCD. The scale bar is 100 μm.

The MRS and MDS model membranes were visualized upon their compression, and the representative BAM images for MRS are presented in Figure 5 and for MDS in Figure S11 in the Supporting Materials.

The undoped MRS membrane was homogeneous at π = 20 and 30 mN/m, whereas at 5 mN/m, multiple separate condensed domains were visible, while in the image taken at 15 mN/m, the process of domain fusion was caught (see Figure 1b). The addition of TBBPA blocks the fusion of the condensed domains and they remain separated even at 30 mN/m. Thus, the BAM pictures depict a biphasic system in which the condensed (LC) domains remain in equilibrium with a thinner continuous phase (the dark region between the domains). The increase of X(TBBPA) from 0.1 to 0.2 leads to the decrease of the diameter of the LC domains from ca. 10 to 5 μm. Moreover, at X = 0.2, multiple 3D aggregates are visible at 30 mN/m (not present at X = 0.1). There is agreement with the BAM data and the π–*A* isotherms and C_S_^–1^–π curves discussed above. At X = 0.1, the minimum in the course of the C_S_^–1^–π curve is shallow, whereas at X = 0.2, it is significant and deep. It was proposed that the minimum in the C_S_^–1^–π curve originates from the 2D–3D phase transition, that is, from the expulsion of the BFR molecules from the model membrane and the formation of the 3D aggregates. Thus, the BAM images prove that the deep minimum in the C_S_^–1^–π curve at X(TBBPA) = 0.2 originates from the expulsion of BFR molecules from the model membrane.

For HBCD, a deep minimum in the C_S_^–1^–π curves is visible at π values just over 10 mN/m. In BAM images at π = 10 mN/m, both at X(HBCD) = 0.1 and 0.2, multiple condensed domains are visible, but no 3D aggregates were observed, whereas at 20 and 30 mN/m numerous bright 3D aggregates dominate the field of view of the microscope. Similarly to TBBPA, the presence of HBCD in the membrane inhibits the fusion of the condensed domains.

For BTBPE at X = 0.1 and π = 10 mN/m, numerous narrow bright filamentous and granular aggregates are visible in the BAM images. With increasing π value, the number of the granular aggregates grows, whereas the number of filamentous domains seems to be constant, oscillating in the view field of the microscope upon the monolayer compression. At X(BTBPE) = 0.2, the number of the granular aggregates is visibly greater. The filamentous domains are blurred and it was impossible to eliminate the interference fringes from the images using the procedure of background correction in BAM software. Thus, it can be inferred that the width of the filamentous domains is resolution-limited, so they are narrower than 2 μm. It should be underlined that the presence of the multilayer 3D aggregates in/on top of the MRS monolayer neither affects the location of the π–*A* isotherms nor changes their courses. Only the lowering of C_S_^–1^ values was observed at higher surface pressures; thus, the presence of these aggregates affects the elasticity of the model membranes. It can be inferred from the lack of shift of the π–*A* isotherms that only a few phospholipid molecules are involved in the formation of the filamentous aggregates, whereas most of the BTBPE molecules separate from the model membrane forming the granular aggregates.

Selected BAM images for the BFR-doped MDS membranes are presented in Figure S13 in the Supporting Materials. Qualitatively, these results are very similar to those discussed above for the MRS model; thus, the discussion and interpretation of the results provided for MRS is also valid for the MDS model membrane.

The BFR-doped MRS and MDS membranes were also studied with the GIXD technique. For TBBPA, such measurements were performed at π = 20 mN/m, as in such conditions, no 3D aggregates are observed in BAM images, and the π–*A* isotherms both at X(TBBPA) = 0.1 and 0.2 are still shifted toward greater mean molecular areas, so it can be assumed that at 20 mN/m TBBPA molecules are still incorporated into the model membranes. The GIXD results for these systems are presented in Figure S14 in the Supporting Materials. It turned out that all the structural parameters extracted from the GIXD measurements for the undoped and TBBPA-doped membranes were comparable. There were, however, changes in the type of the 2D lattice type. For MRS at π = 20 mN/m, the GIXD data were best fitted by two Lorentz curves, so the lattice was rectangular centered. In the presence of TBBPA, the data were best fitted with three Lorentz curves, so the lattice was oblique. For MDS at π = 20 mN/m, the packing of hydrocarbon chains was described by a rectangular centered lattice and was unchanged after the introduction of TBBPA into the model membrane. The intensities of the diffraction signal for the undoped membranes and TBBPA-doped were also comparable. This means that the addition of TBBPA does not lead to the lowering of the number of 2D crystalline nanodomains diffracting the X-rays. Based on the identical structural parameters extracted from the GIXD data for the TBBPA-doped and undoped monolayers, it can be stated that TBBPA molecules do not incorporate into the 2D crystalline domains in the MRS and MDS membranes. Thus, the question arises what happens with the TBBPA molecules? They do not form 3D aggregates and thus should be built into the monolayers; however, they are not included in the 2D crystalline membranes. The answer to this question comes after analyzing BAM images. The presence of TBBPA molecules inhibits the fusion of condensed domains both in the MRS and MDS membranes and preserves the monolayer regions in the liquid expanded state until high surface pressure values. Thus, it can be assumed that TBBPA molecules are incorporated into the continuous (LE) regions of the monolayer. In our previous studies, similar conclusions were drawn for cyclodiene pesticides incorporated into model fungal membranes.^63^ The presence of TBBPA affects slightly the lateral pressure in the studied monolayers, and this changes the equilibrium between the condensed domains and the continuous phase. These changes can lead to the observed fluctuations in the value of the tilt angle, i.e., a 1.1° decrease for MRS at X(TBBPA) = 0.1 and a 1.7° increase for MDS at X(TBBPA) = 0.2.

The HBCD-doped MRS and MDS membranes were also studied with the application of the GIXD technique. The GIXD results are presented in Figure S13 in the Supporting Materials. Again, similar to TBBPA, the GIXD data collected for the HBCD-doped membranes were identical within the experimental error with those collected for the undoped monolayers. For HBCD-doped monolayers, the long plateau in the course of the π–*A* isotherms (see Figure 4) begins just above π = 10 mN/m. Thus, first, the GIXD experiments were performed at 10 mN/m, and then these measurements were repeated at 20 mN/m, that is above the plateau region, at a surface pressure where the isotherms for the HBCD-doped and undoped monolayers are located close to each other. The identical parameters extracted from the GIXD data for the HBCD-doped and undoped monolayers confirm that HBCD molecules do not incorporate into the 2D crystalline domains. Again, HBCD inhibits the fusion of the domains and preserves the regions of the monolayer in the LE state; thus, similar to TBBPA, it can be assumed that below the transition surface pressure (ca. 10 mN/m), the HBCD molecules are incorporated into the LE regions of the monolayer. The increase in surface pressure leads to the expulsion of the HBCD molecules from the model membrane and the nucleation of 3D aggregates.

**Figure 6:**
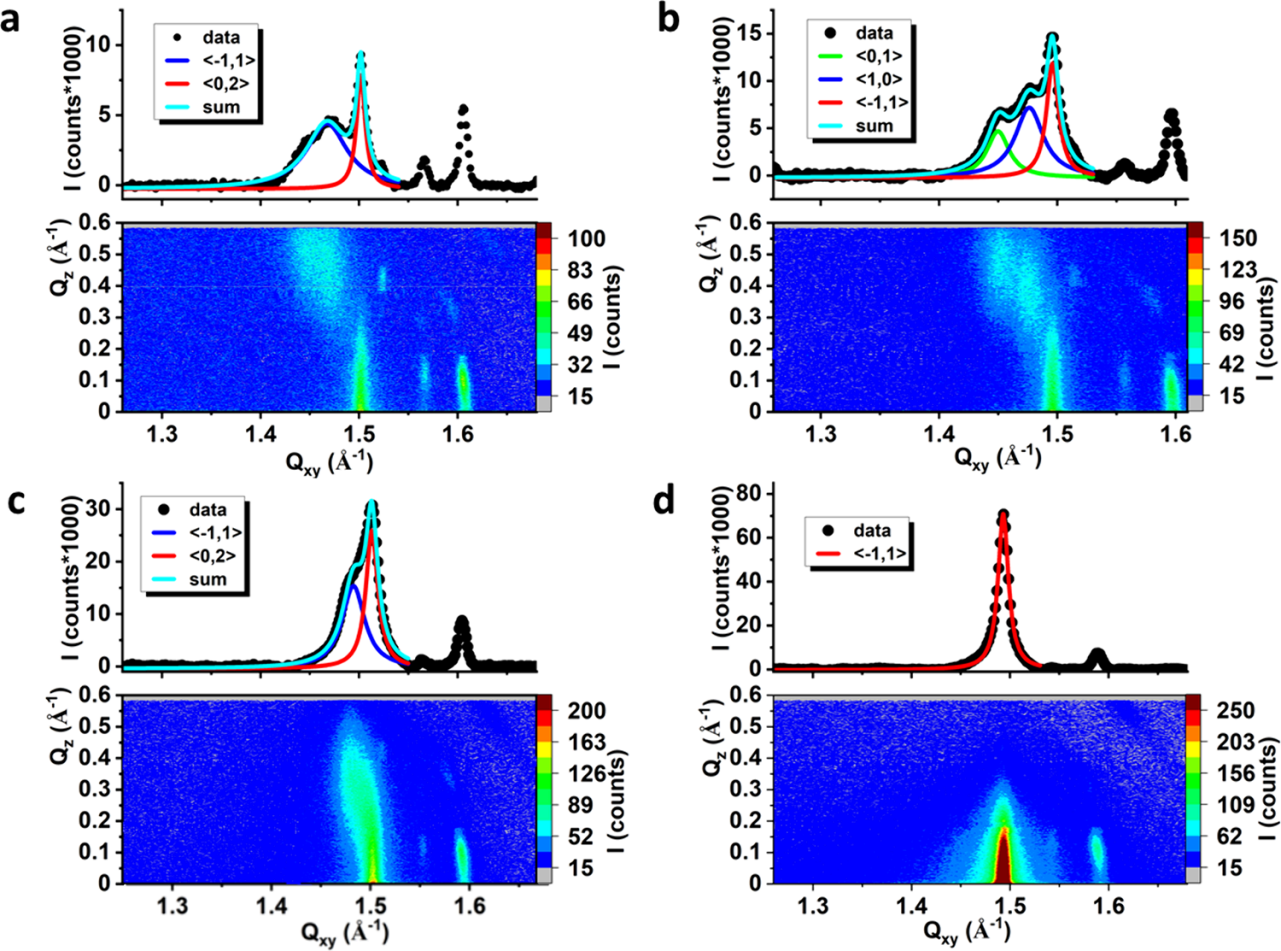
GIXD data for the BTBPE-doped model membranes, X(BTBPE) = 0.1. (a) MRS, π = 10 mN/m, (b) MDS, π = 10 mN/m, (c) MDS, π = 20 mN/m, (d) MDS, π = 30 mN/m. Solid lines in the Bragg peak profiles are Lorentz curves fitted to the experimental data.

GIXD measurements were also performed for the BTBPE-doped membranes and the results are presented in Figure 6, while the structural parameters extracted from the GIXD data (calculated from the peaks originating from the X-ray diffraction on monolayer structures) are summarized in Table S3 in the Supporting Materials.

**Figure 7:**
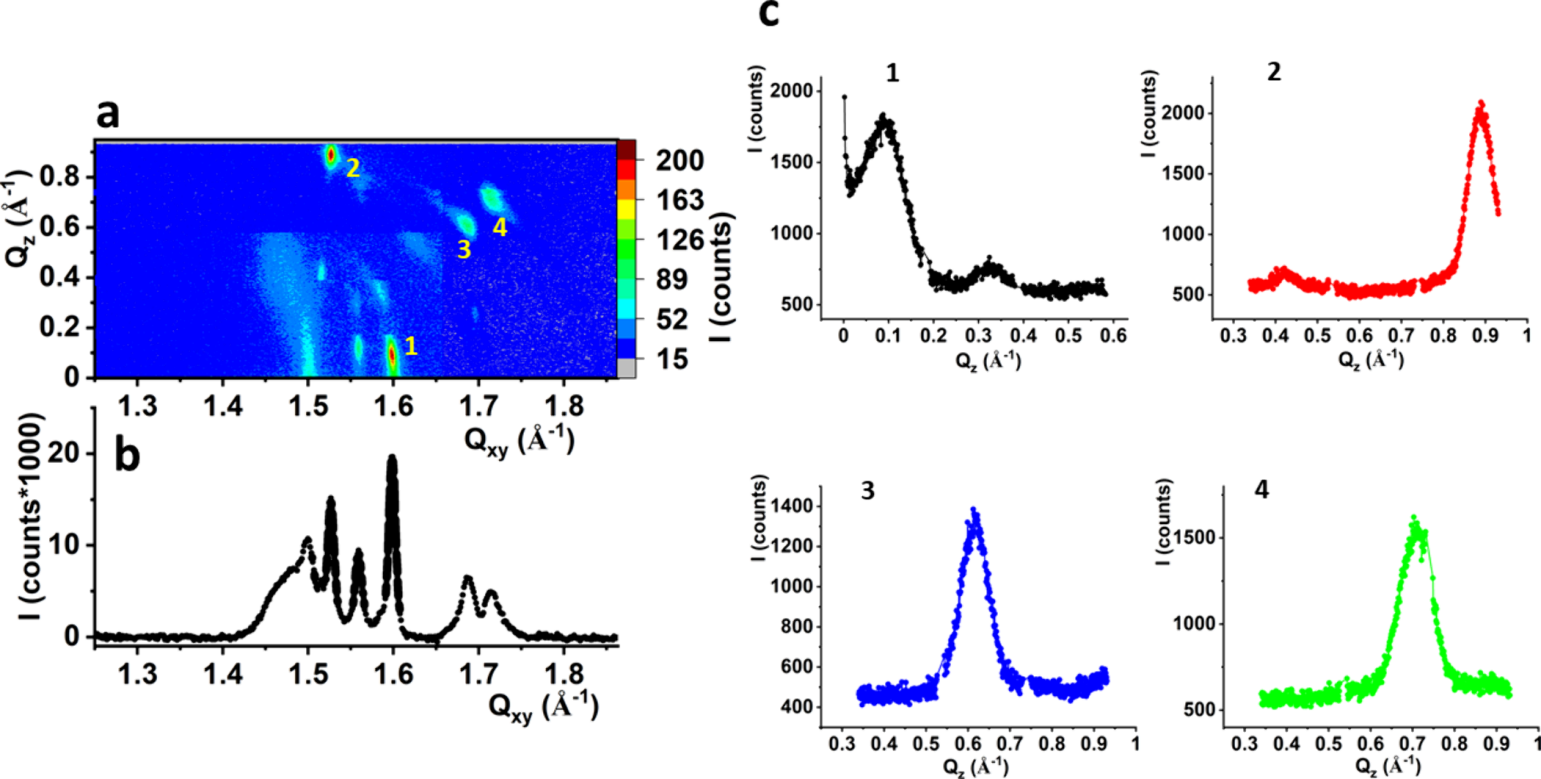
GIXD data for the BTBPE-doped MDS membrane, at X(BTBPS) = 0.2 and π = 10 mN/m: (a) I(Q_*xy*_,Q_*z*_) contour intensity map, (b) I(Q_*xy*_) Bragg peak profile, and (c) I(Q_*z*_) Bragg rod profiles calculated for the diffraction signals numbered from 1 to 4.

At π = 10 mN/m in the intensity contour maps I(Q_*xy*_,Q_*z*_) recorded for the BTBPE-doped MRS and MDS model membranes numerous diffraction peaks can be seen. They can be divided into two categories: the wider intense peaks located at Q_*xy*_ ranging from 1.4 to 1.5 Å^–1^ and narrow less intense peaks located at Q_*xy*_ > 1.5 Å^–1^. The wider peaks are identical to those measured for the undoped model membranes (see Figure 2), thus originating from the X-ray diffraction on phospholipid monolayers. These peaks were used to calculate the structural parameters for these monolayers (Table S3). Similar to TBBPA and HBCD, the addition of BTBPE also does not affect the values of lattice parameters, the molecular tilt, or the range of 2D crystallinity within the monolayer plane. Thus, also for BTBPE, it can be stated that the molecules of this BFR do not incorporate into the 2D crystalline monolayer domains. However, the introduction of BTBPE to both MRS and MDS monolayers leads also to the appearance of narrow, less intense diffraction peaks: a stronger peak with its intensity at Q_*xy*_ = ca. 1.6 Å^–1^ and Q_*z*_ = 0.1 Å^–1^, and also weaker peaks (Q_*xy*_ = 1.52 Å^–1^, Q_*z*_ = 0.42 Å^–1^; Q_*xy*_ = 1.57 Å^–1^, Q_*z*_ = 0.12 Å^–1^). Bragg peak profiles calculated for these maxima are very narrow, which suggests an X-ray diffraction on a multilayer object. The presence of these additional peaks indicates that the filamentous structures visible in BAM images for the BTBPE-doped MRS and MDS membranes are built of periodically organized molecules. The intensity of the diffraction signal originating from the diffraction on the monolayer (nanodomains of one-molecule thickness) is much greater than the intensity of peaks originating from the diffraction on multilayer structures. The data presented in Table S3 confirm that the addition of BTBPE does not affect the packing of the phospholipid molecules in the 2D crystalline one-molecule-thick nanodomains within the monolayer. Thus, in agreement with the above-discussed π–*A* isotherms, the incorporation of BTBPE molecules into the model bacterial membranes is very limited; however, those few molecules that are incorporated into the monolayer can induce the formation of the multilayer filamentous structures. The BTBPE-doped MDE membranes were also compressed to 20 and 30 mN/m (Figure 6 c,d). The rise in surface pressure increases the intensity of the diffraction signal ascribed to the monolayer, whereas the intensity of the signal ascribed to the multilayer domains remains practically unchanged. This is in agreement with the observations made during the microscopic studies (see Figure 5). With rising π, the number of granular aggregates increased, but the number of filamentous remained virtually unchanged. Thus, in the next step of our research, we increased the mole ratio of BTBPE in the MDS membrane to 0.2 and repeated the measurements at π = 10 mN/m. The GIXD data for this experiment are presented in Figure 7.

The GIXD data for X(BTBPE) = 0.2 have been collected for a wider range of Q_*xy*_ and Q_*z*_. The increase in the BTBPE mole ratio leads to more diffraction peaks. The signals located at the Q_*xy*_ range from 1.4 to 1.5 Å^–1^ originate from the diffraction of X-rays on the domains of monomolecular thickness, whereas all the other peaks result from the diffraction on multilayer structures. Now at X = 0.2, the intensity of the signals related to multilayer is much greater than at X = 0.1. Four peaks ascribed to the multilayer structures numbered in the intensity contour map from 1 to 4 have a relatively high intensity. Thus, for them, it is possible and reasonable to calculate the Bragg rod profiles, that is, the dependence I(Q_*z*_) integrated over Q_*xy*_. The Bragg rod profiles for the maxima 1 to 4 are presented in Figure 7c. According to the Scherrer formula,^71,72^ the full width at half-maximum (fwhm) of a Bragg rod can be used for the calculation of the thickness of the layer coherently diffracting X-rays.
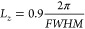


**Scheme 2:**
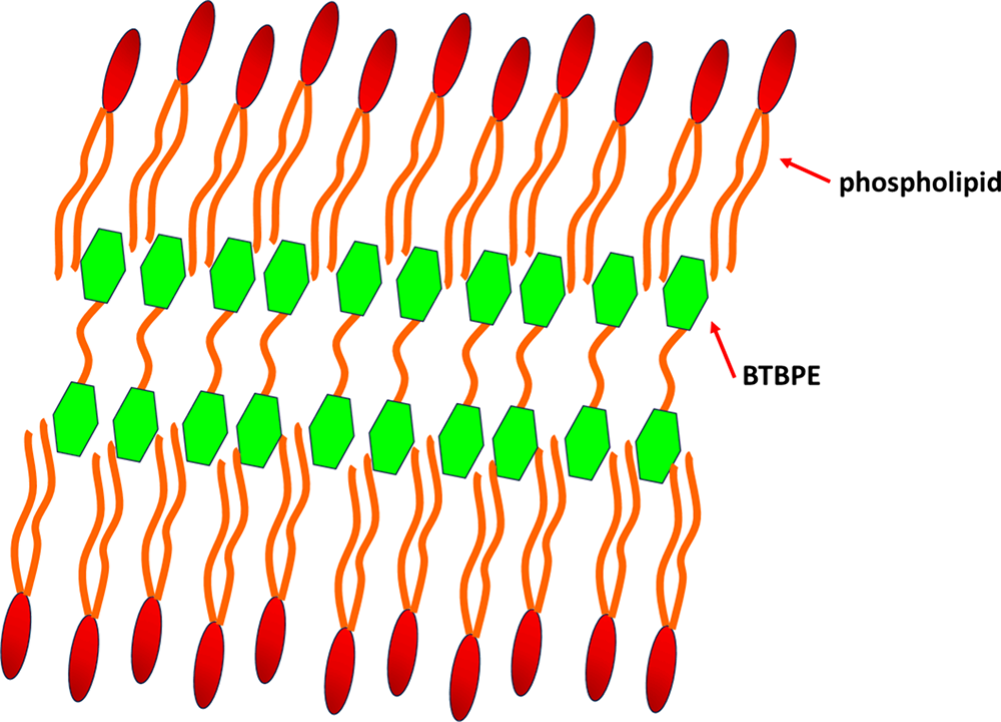
Possible Packing of the Phospholipid and BTBPE Molecules in the Crystalline Multilayers

For Bragg rods 1 to 4, the fwhm’s are 0.058, 0.067, 0.068, and 0.074, respectively. This leads to the following values of L_*z*_: 97.5 Å; 84.4 Å, 83.2 Å, and 75.4 Å, respectively. The length of the hydrocarbon chain of DPPG and DPPE can be calculated according to Tanford’s formula: L = 1.265n + 1.50 (Å),^73^ where n is the number of carbon atoms in the hydrocarbon chain. Assuming that the carbon atom of the carboxyl group belongs to the headgroup, n = 15 for the palmitoyl chain, thus, L = 20.5 Å. The length of the headgroup can be estimated as ca. 10 Å.^74^ Thus, the whole length of the phospholipid molecules used in the MDS model is ca. 30 Å. The length of BTBPE is ca. 15–20 Å depending on the conformation of this molecule. Therefore, the thickness of the multilayer estimated between 75.4 to 97.5 Å can correspond to two phospholipid monolayers, between which the BTBPE molecule is sandwiched. An illustration of the proposed organization of the multilayer is depicted in Scheme 2. In this scheme, we tried to include the tilt of the molecules as well as the undulation of the bilayer, which consequently leads to the increase of its effective thickness. The values of ca. 83–84 Å estimated from the fwhm of peaks 2 and 3 seem here as a reasonable thickness for such a structure. Opting for the bilayer structure of the filamentous aggregates, another feature of the GIXD data should also be taken into consideration. The Bragg rods for the most intense multilayer diffraction peaks 1 and 2 are bimodal. For peak number 1, the main maximum at Q_*z*_ of ca. 0.1 Å^–1^ is accompanied by a weaker maximum at Q_*z*_ = 0.3 Å^–1^. Similarly, for the peak number 2, the main maximum at Q_*z*_ = 0.88 Å^–1^ is accompanied by a weaker one at Q_*z*_ = 0.42 Å^–1^. The presence of intensity modulations of the Bragg rods along Q_*z*_ is typical of multilayer structures.^75^ For example, for a bilayer formed in a collapsed cholesterol Langmuir monolayer, the Bragg rods are bimodal.^76,77^ Thus, for our research, the bimodal Bragg rods for peaks 1 and 2 confirm that the structures induced by the presence of BTBPE in a phospholipid monolayer are bilayer-thick. It is also reasonable to ask why it is BTBPE and not another of the studied BFRs that causes the formation of the observed multilayer structures. A glance at Scheme 1 shows that TBBPA and BDE 99 are structurally similar to BTBPE. However, these three molecules differ in the structure of the spacer joining the two benzene moieties. In TBBPA, it is a bulky −C(CH_3_)_2_– group, in BDE 99, just an ether oxygen atom, while in BTBPE, it is a four-atom-long −O–CH_2_–CH_2_–O– fragment. This spacer makes the BTBPE molecule longer and more flexible than the TBBPA and BDE 99 molecules, which can be crucial in the efficient fitting of this molecule to the hydrocarbon chains of phospholipids. In TBBPA, the bulky −C(CH_3_)_2_– group can be the steric hindrance disabling such a fit, while the BDE 99 molecule is just too short.

**Figure 8:**
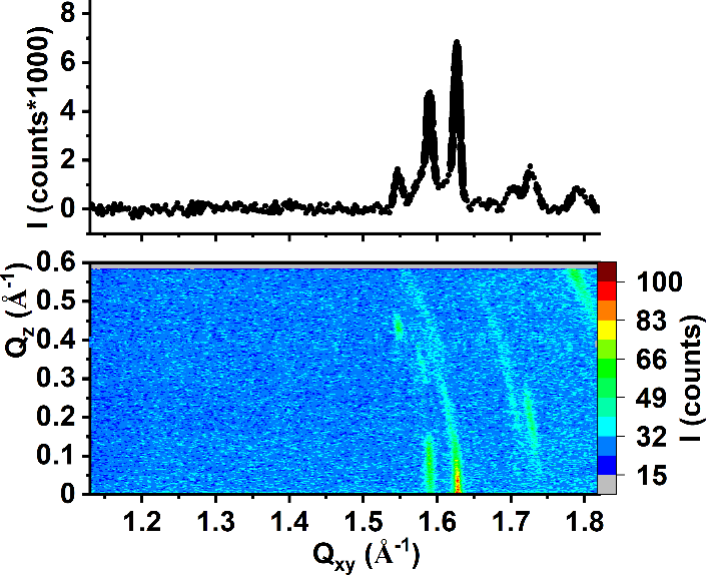
GIXD data: I(Q_*xy*_,Q_*z*_) contour intensity map and I(Q_*xy*_) Bragg peak profile for the BTBPE-doped POPE monolayer. X(BTBPE) = 0.1, π = 10 mN/m.

We also repeated the GIXD experiments for the BTBPE-doped MRU and MDU model membranes, that is, models formed from the unsaturated dioleoyl lipids, as for these models also the filamentous multilayer structures were observed in BAM images. In these measurements, no diffraction signal was observed. No diffraction signal was also observed for one-component DOPC, DOPE, and DOPG monolayers doped in BTBPE. To find out how the phospholipid structure affects the crystallinity of the sandwiched bilayers formed between phospholipid molecules and BTBPE, we also applied GIXD to study the interactions between POPE and BTBPE molecules. sn1-saturated sn2-unsaturated phospholipids are widespread in bacterial membranes,^78^ so such a study could shed additional light on the BTBPE-bacterial membrane interactions. Moreover, for a BTBPE-doped POPE monolayer, the filamentous structures were also observed – see Figure S16 in the Supporting Materials. Below in Figure 8, the GIXD data collected in this experiment are presented.

The measurements were performed at π = 10 mN/m, as at this surface pressure, the POPE monolayer spread on water is in the LE state,^79^ and no X-ray diffraction signal is observed. When the monolayer was doped in BTBPE, the diffraction pattern shown in Figure 8 was recorded. First, it should be underlined that none of the diffraction peaks in Figure 8 originates from the diffraction on a phospholipid monolayer. For a typical phospholipid monolayer, the diffraction signals cluster between 1.4 and 1.5 Å^–1^, and in this fragment of the intensity map, there is no signal at all. Thus, all the diffraction signals measured in this experiment originate from the diffraction of X-rays on multilayer structures. The intensity of the narrow diffraction signals is distributed on the characteristic Scherrer arcs.^80^ It means that the crystalline nanodomains are randomly oriented at the air/water interface and that there is no one fixed tilt angle of the palmitoyl chains, but it changes between the domains. As there was no diffraction signal for the BTBPE-doped DOPE monolayer, it can be inferred that for the BTBPE-doped POPE monolayer, only the palmitoyl chains are periodically ordered in the BTBPE-induced multilayer domains. This together with the unfixed tilt of these chains leads to low intensity of the diffraction signal, which disables the calculation of L_*z*_ from Bragg rods and by this the estimation of the thickness of the BTBPE-induced domains in the POPE monolayer.

## Conclusions

Seven BFRs very often used in plastic production and known as emerging soil contaminants were studied. It turned out that these compounds differ significantly in membrane activity. The incorporation of small polybrominated benzene derivatives such as TBP and in particular PBT to the model membranes was limited. In real conditions, this may mean difficulties in the uptake and metabolism of these substances by the dehalogenating soil bacteria. On the other hand, the incorporation of TBBPA, BDE 99, and the two polybrominated cycloalkanes TBCO and HBCD was easier. At the level of 10 mol %, these substances were entirely incorporated into the model membranes, especially those prepared from unsaturated phospholipids. The further increase in the concentration of these BFRs leads to the formation of 3D aggregates, which indicates that several mole % is the impassable level of the incorporation of these BFRs into the applied model membranes. Among the polybrominated cycloalkanes, HBCD had a much greater influence on the model membranes than TBCO. HBCD incorporated more easily into the model membranes than TBCO, achieving higher mole ratios. However, too much accumulation of HBCD affects the membrane elasticity, which in real membranes could affect their fluidity and permeability, and therefore disturb their function. Thus, the ban on HBCD and replacing it with TBCO seems reasonable. To get a better insight into the BFR-membrane interactions, we also used the models prepared solely from saturated phospholipids. The use of these models allowed us to demonstrate that BFR molecules partition into the liquid expanded regions of the monolayer and avoid condensed domains. In our models, the presence of BFR molecules preserves these LE regions and limits the fusion of the condensed domains. The exclusion of BFR molecules from condensed monolayer domains is an interesting result, as it can mean that in real membranes, BFRs may also avoid the stiffer regions of the membrane. The most interesting results were obtained for BTBPE. The production of this novel BFR is increasing, as it replaced the banned PBDEs on the market. At first glance, judging only by the course of the π–*A* isotherms and C_S_^–1^–π, the incorporation of this compound into the membranes is limited and the incorporated molecules do not affect noticeably the physical properties of the model membranes. However, in BAM images bright filamentous domains were observed in BTBPE-doped membranes. Their morphology was similar both in the monolayers formed of unsaturated as well as of saturated phospholipids. The application of the GIXD technique indicated that in monolayers formed of saturated or mixed-chain phospholipids, these filamentous aggregates are crystalline, whereas in the membranes formed of unsaturated phospholipids, these structures were amorphous. The thorough analysis of the GIXD data indicated that these filamentous domains have a bilayer thickness. Thus, we proposed a model of this bilayer in which the BTBPE molecules are sandwiched between two layers of tilted phospholipid molecules. The formation of such extrusions in real membranes could be fatal for bacterial cells. Unfortunately, the number of articles regarding the bioremediation of BTBPE-contaminated soil is very limited; thus, the toxicity of BTBPE molecules to soil microorganisms is widely unknown. This gap should be filled in the future by research on bacterial cultures, to verify our results established for model systems. Our research proves that the use of simple models of biological membranes such as Langmuir monolayers can be a quick and effective way to test new BFRs in terms of their potential microbial toxicity and biodegradability.

## Supporting Information Available

π–*A* isotherms and C_S_^–1^–π curves for one-component monolayers formed of the phospholipids used for the model membrane preparation; π–*A* isotherms and C_S_^–1^–π curves for the BFR-doped MRU, MDU, MRS, and MDS model membranes; selected BAM images for the BFR-doped MRU and MDS model membranes; GIXD data for the TBBPA and HBCD-doped MRS and MDS model membranes; tables with structural parameters calculated from the GIXD data (PDF)
