# Supplementary material for: Interactions of Brominated Flame Retardants with Membrane Models of Dehalogenating Bacteria: Langmuir Monolayer and Grazing Incidence X-ray Diffraction Studies
Source: Langmuir. 2024 May 9;40(20):10600–14. doi: 10.1021/acs.langmuir.4c00518 (PMC11112749; doi:10.1021/acs.langmuir.4c00518)
Supplement: Supplementary file 1 — la4c00518_si_001.pdf [file la4c00518_si_001.pdf]

Supporting Materials

Interactions of Brominated Flame Retardants with Membrane Models of Dehalogenating Bacteria. Langmuir Monolayer and Grazing Incidence X-ray Diffraction Studies

Marcin Broniatowski[1*] and Paweł Wydro[2]

[1] Department of Environmental Chemistry, Faculty of Chemistry, the Jagiellonian University in Kraków, ul. Gronostajowa 2, 30-387 Kraków, Poland

broniato@chemia.uj.edu.pl

[2] Department of Physical Chemistry and Electrochemistry, Faculty of Chemistry, the Jagiellonian University in Kraków, ul. Gronostajowa 2. 30-387 Kraków, Poland

1. **Characterization of the one-component Langmuir monolayers formed from the phospholipids used for the model membrane construction**

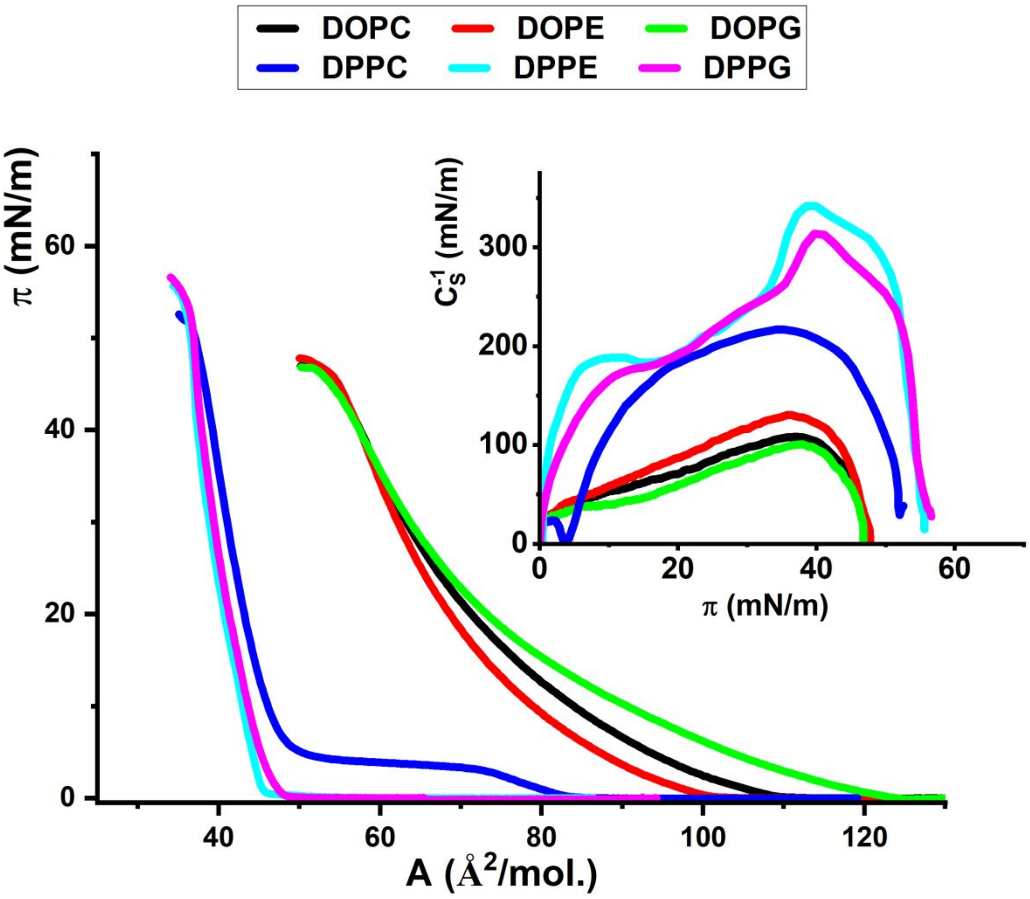

Figure S1. π-A isotherms and C_S^{-1}-π curves measured for one-component Langmuir monolayers formed from the studied phospholipids.

2. Additional data for the MRU model membrane doped in the studied BFRs

2.1. π-A isotherms and C_S^{-1}-π curves

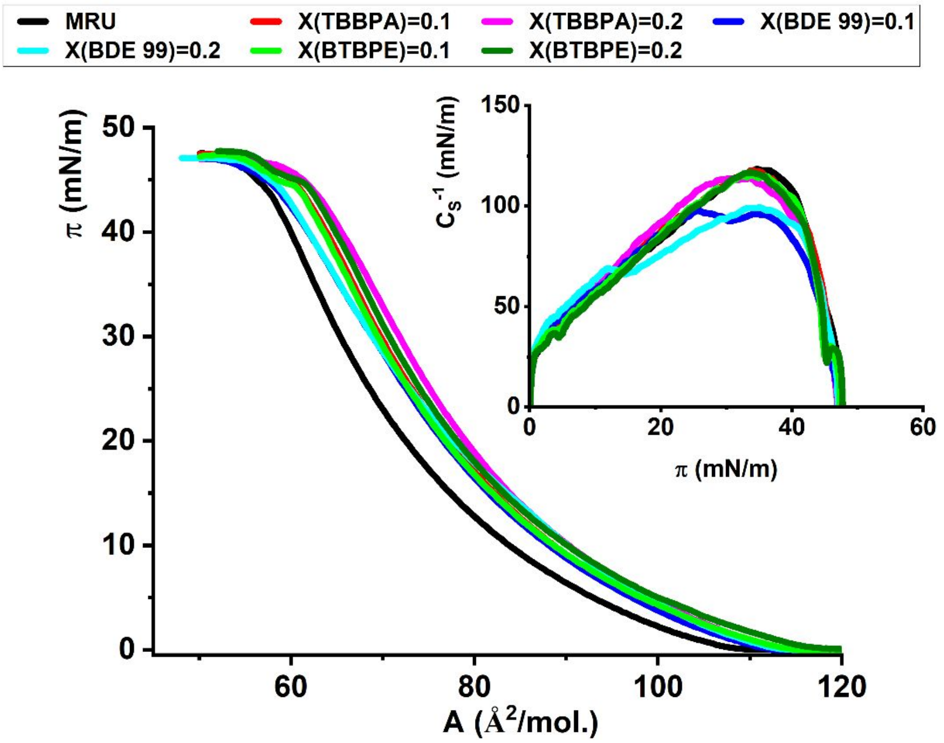

Figure S2. π-A isotherms and C_S^{-1}-π curves for the MRU model membranes doped in TBBA, BDE 99, and BTBPE.

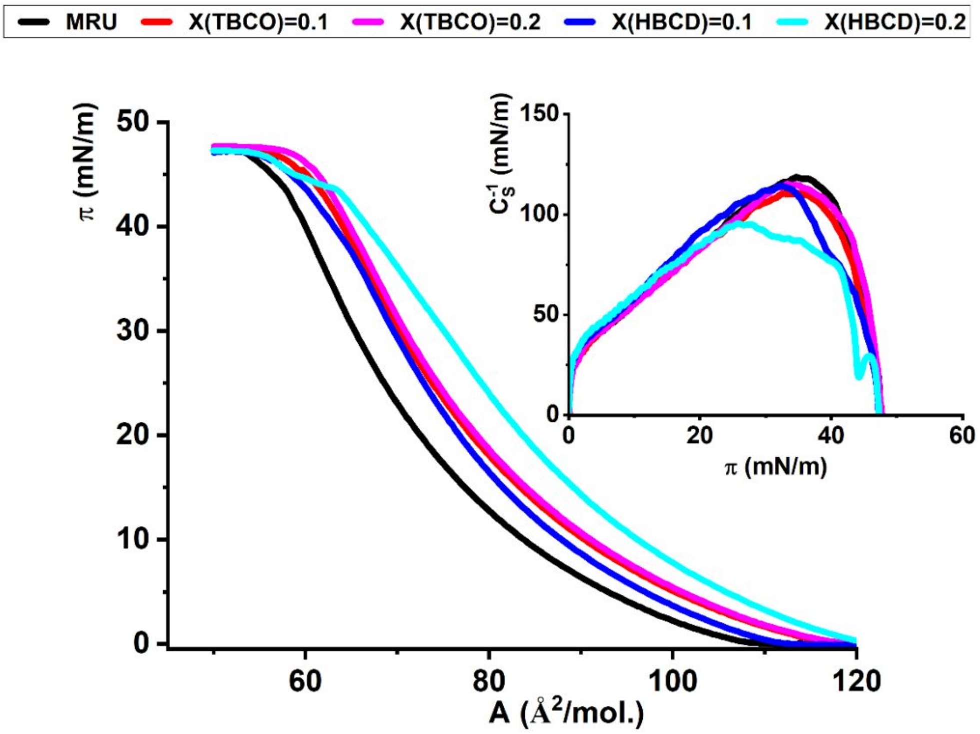

Figure S3. π-A isotherms and C_S^{-1}-π curves for the MRU model membranes doped in TBCO and HBCD.

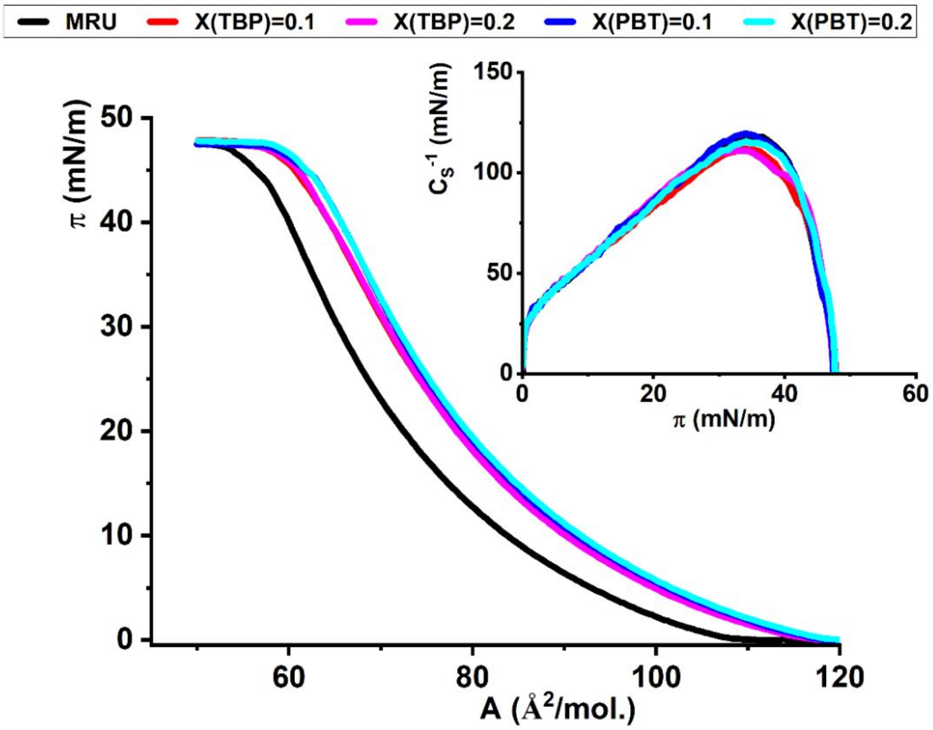

Figure S4. π-A isotherms and C_S^{-1}-π curves for the MRU model membranes doped in TBP and PBT.

2.2. Selected BAM images for the BFR-doped MRU model membranes

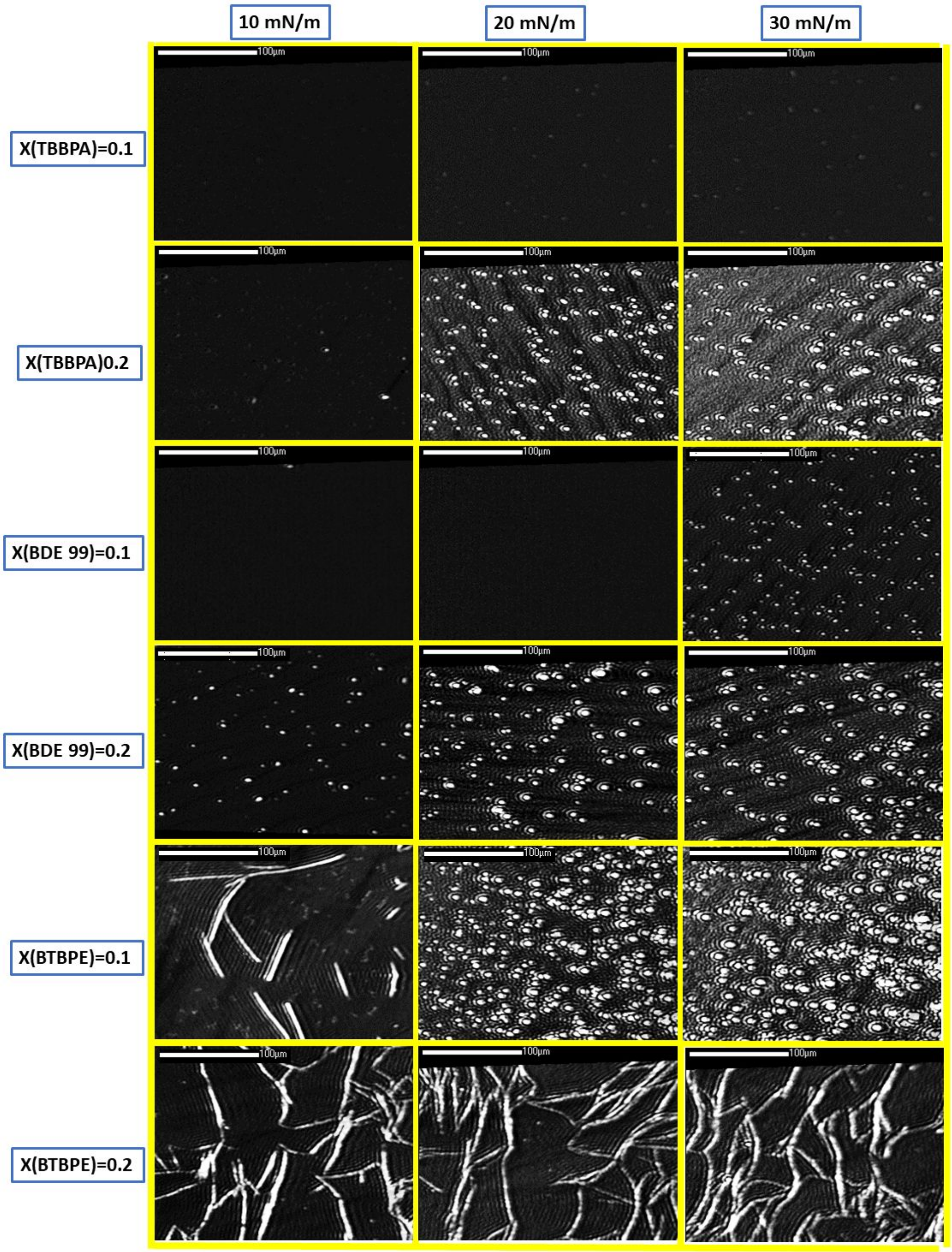

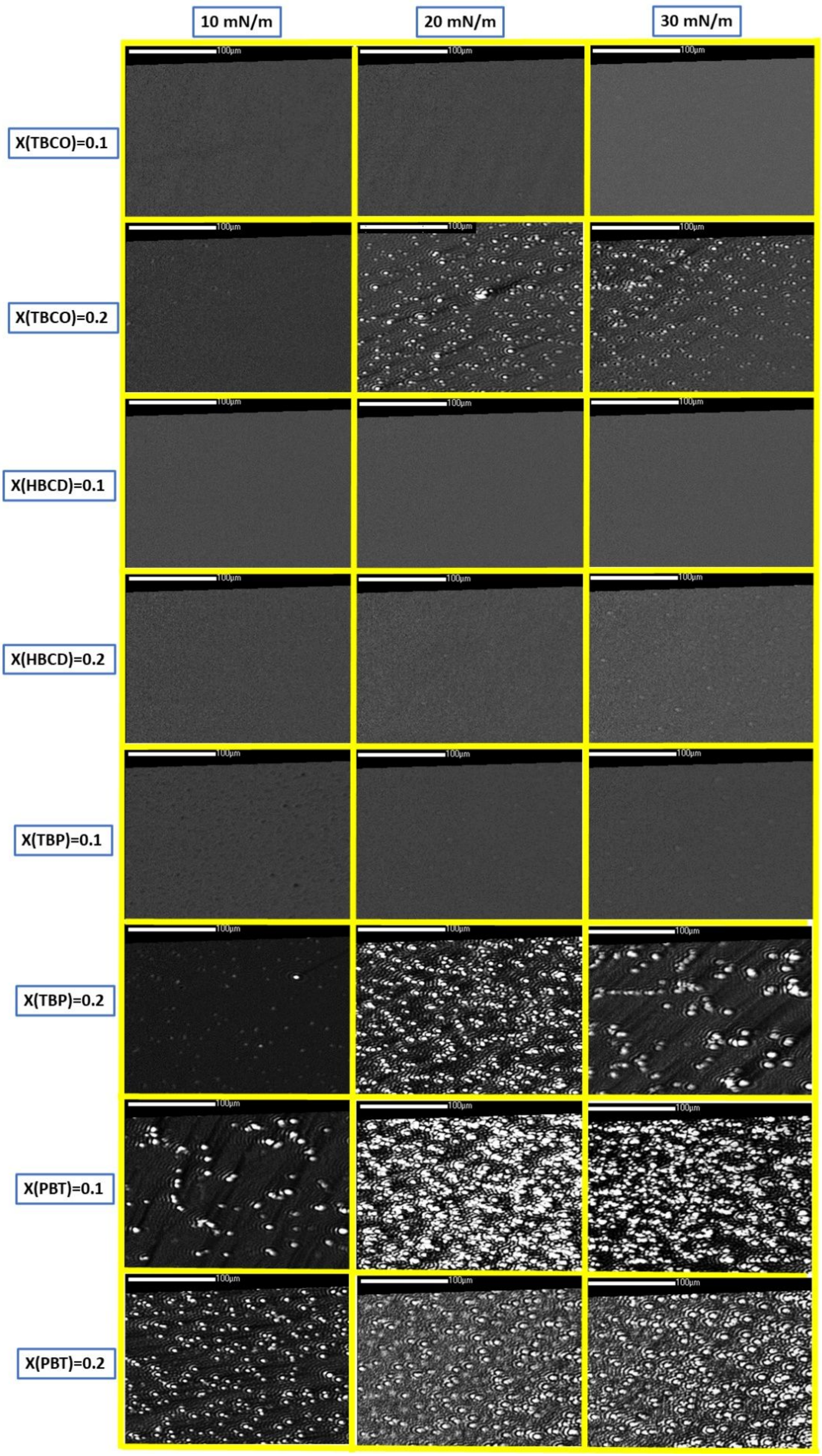

Figure S5. Selected BAM images for the MRU model membranes doped in the studied BFRs. The scale bar depicts 100 μm.

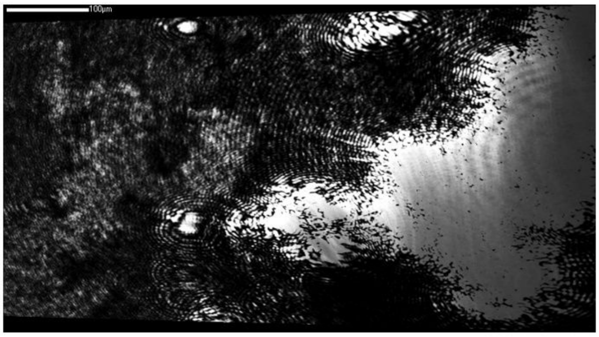

Figure S6. BAM image of the air/water interface after the deposition of BTBPE chloroform solution at the air/water interface and the compression to the most closed position of the barriers, corresponding to the mean molecular area of 20 Å^2 per BTBPE molecule and π = 1 mN/m.

3. Additional data for the MDU model membrane doped in the studied BFRs

3.1. π-A isotherms and C_S^{-1}-π curves

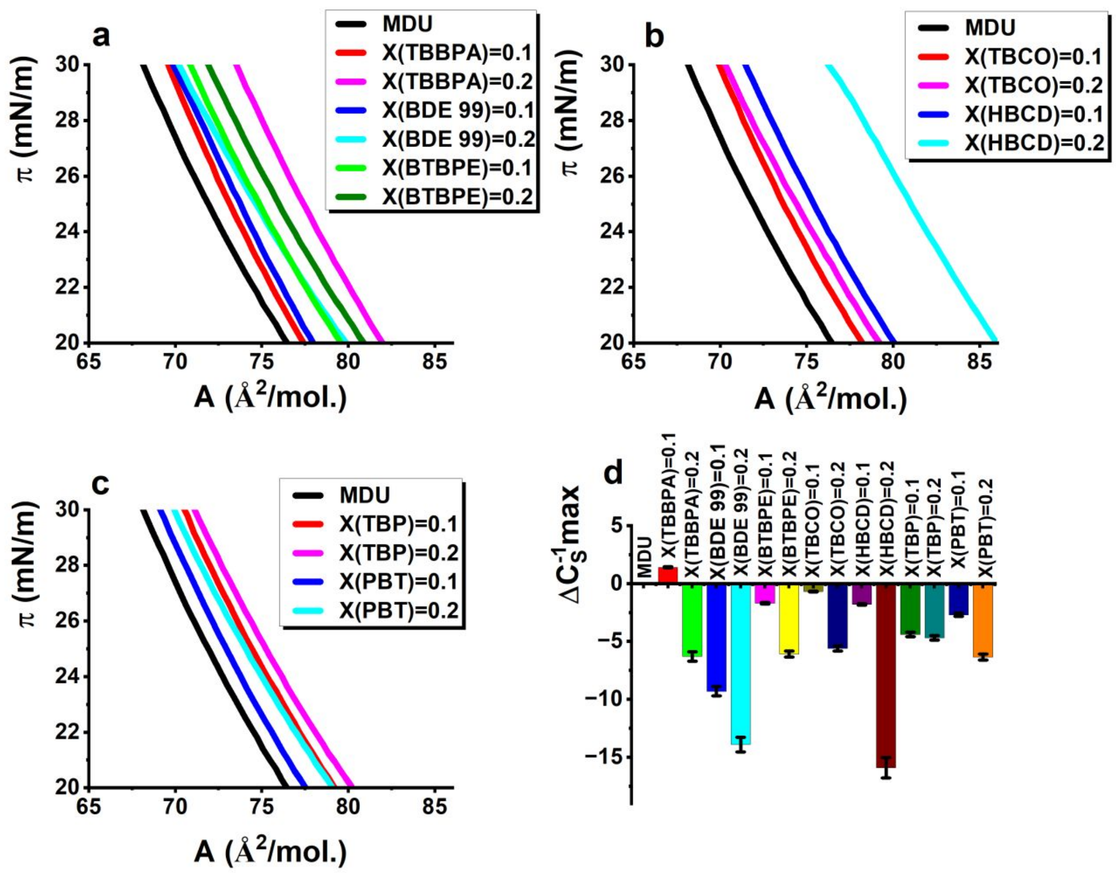

Figure S7. a-c) π-A isotherms (sections from π = 20 to 30 mN/m) for the MDU monolayers doped in a) TBBPA, BDE 99, BTBPE, b) TBCO and HBCD, c) TBP and PBT. d) difference between the maximal C_S^{-1} values observed for a doped monolayer and the MDU model membrane.

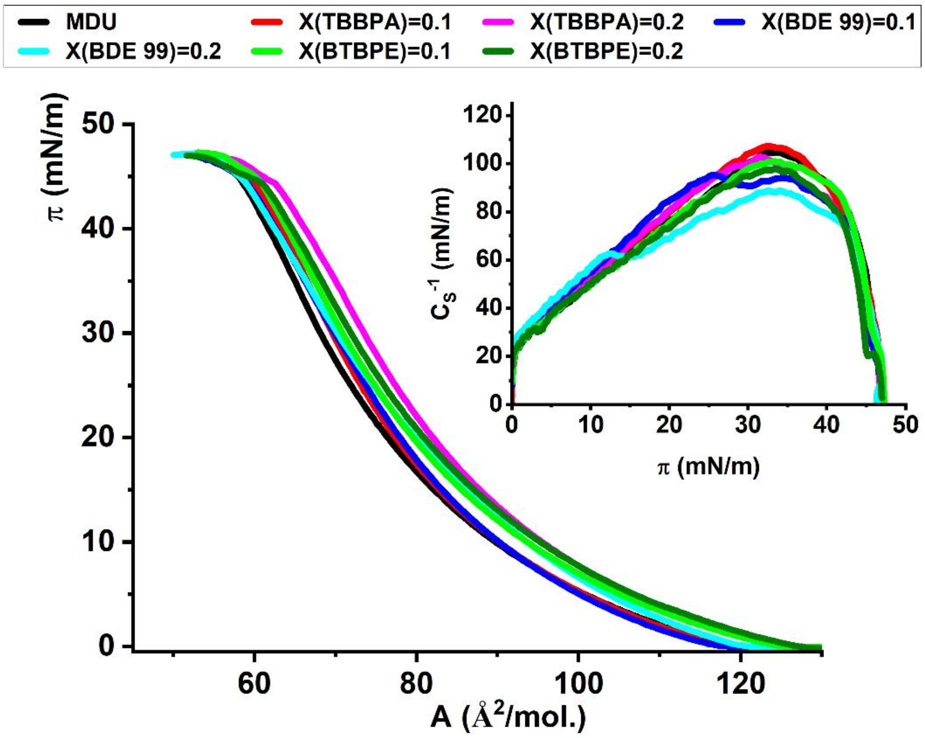

Figure S8. π-A isotherms and C_S^{-1}-π curves for the MDU model membranes doped in TBBA, BDE 99, and BTBPE.

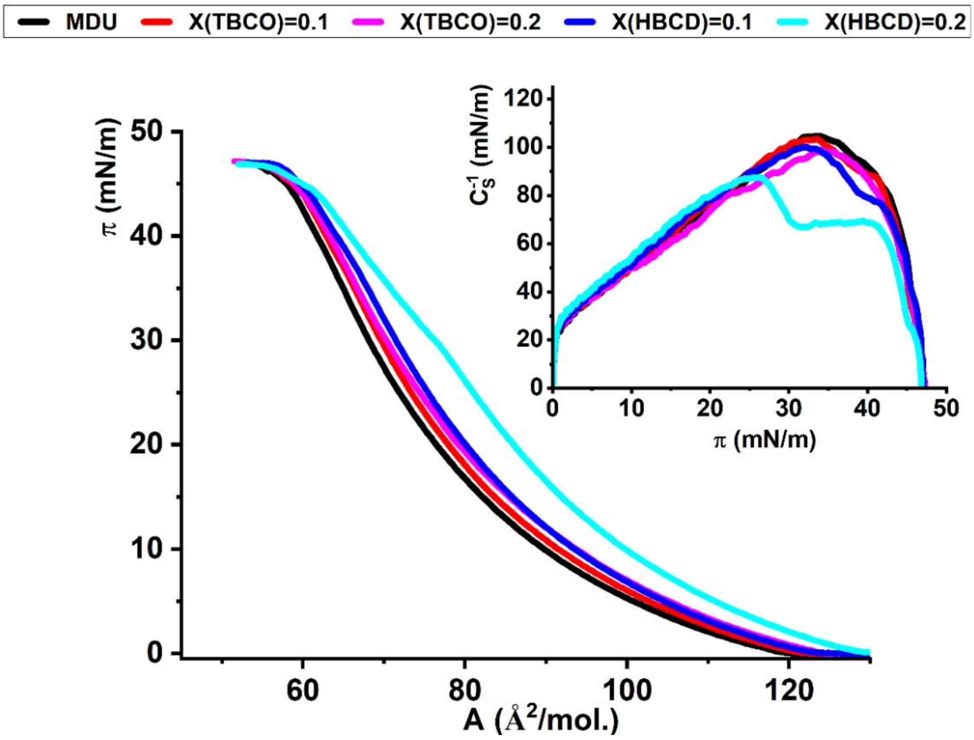

Figure S9. π-A isotherms and C_S^{-1}-π curves for the MDU model membranes doped in TBCO and HBCD.

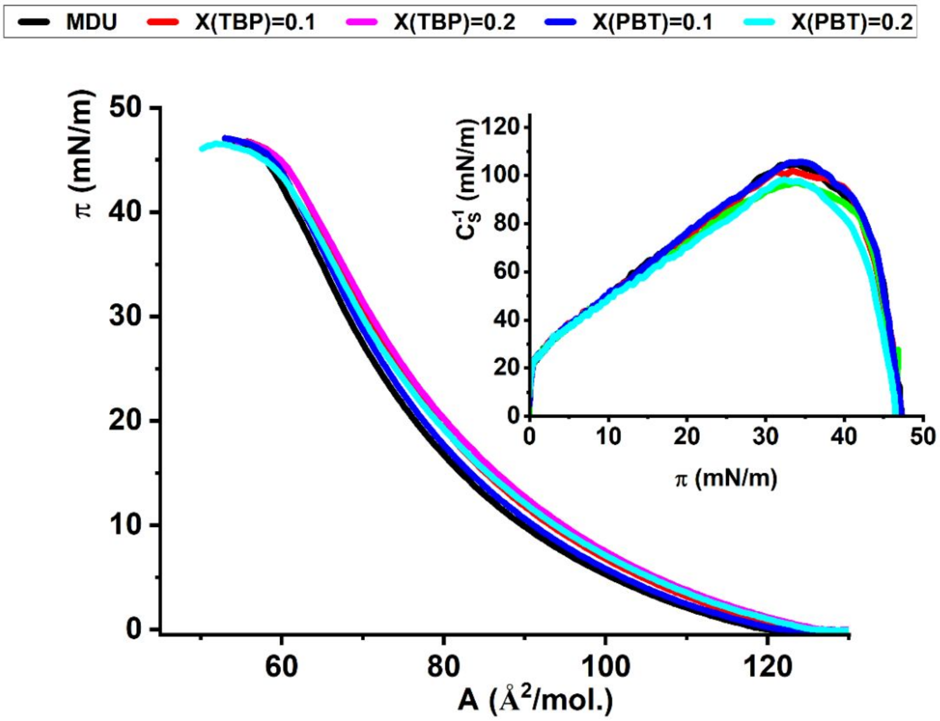

Figure S10. π-A isotherms and C_S^{-1}-π curves for the MDU model membranes doped in TBP and PBT.

4. Additional data for the MRS and MDS model membranes doped in the studied BFRs

4.1. π-A isotherms and C_S^{-1}-π curves

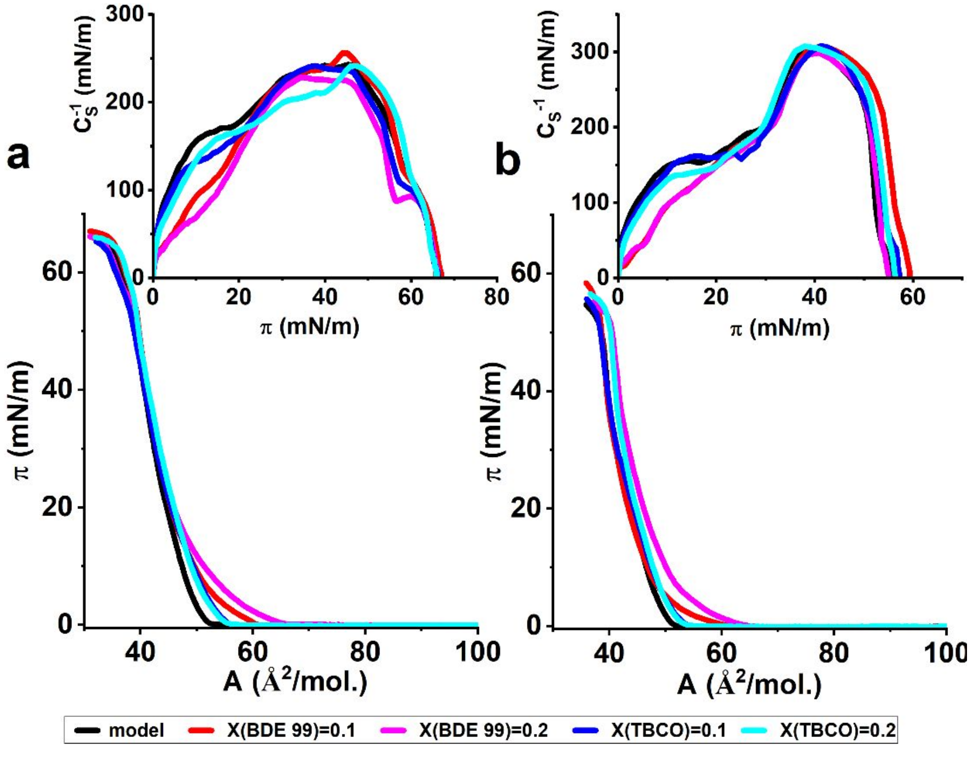

Figure S11. π-A isotherms and C_S^{-1}-π curves for a) MRS, and b) MDS model membranes doped in BDE 99 and TBCO.

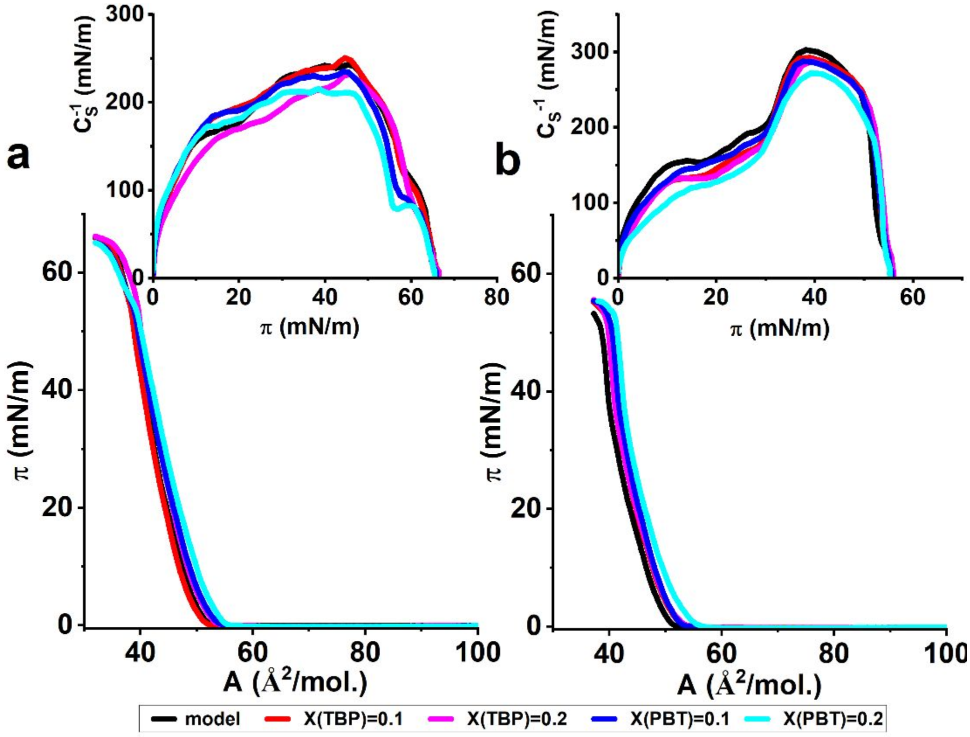

Figure S12. π-A isotherms and C_S^{-1}-π curves for a) MRS, and b) MDS model membranes doped in TBP and PBT.

4.2. Selected BAM images for the BFR-doped MDS model membranes

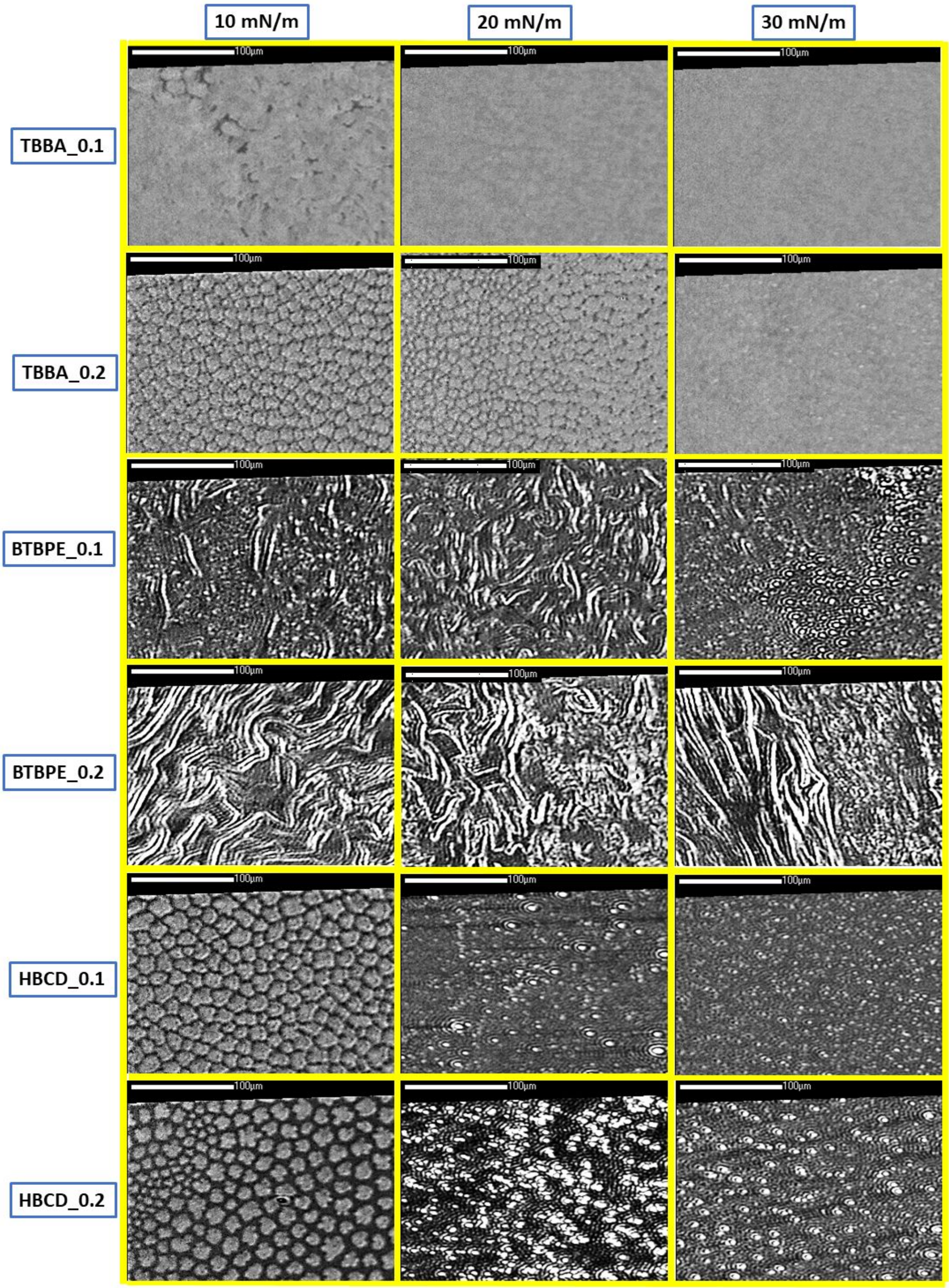

Figure S13. Selected BAM images for the MDS model membrane doped in TBBA, BTBPE, and HBCD.

4.3. GIXD results for the MRS and MDS model membranes doped in TBBPA and HBCD

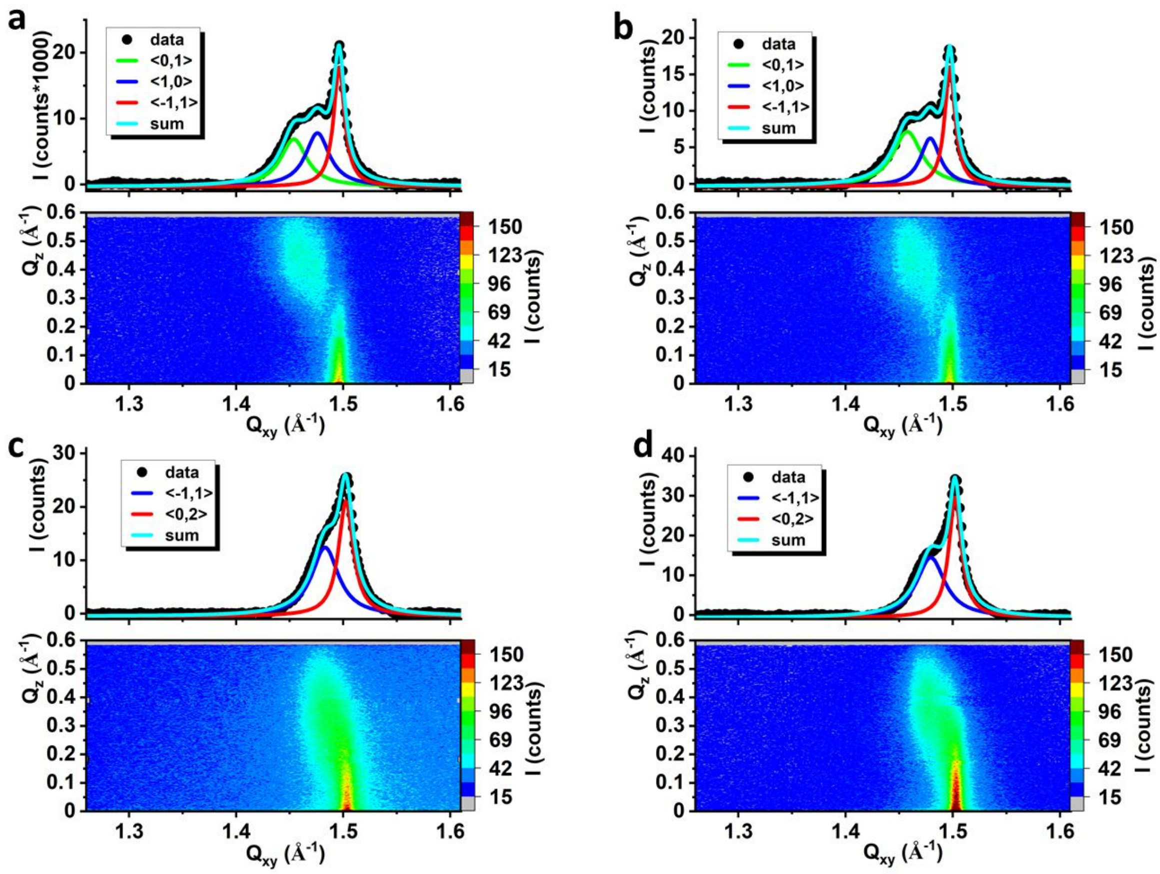

Figure S14. GIXD results: I(Q_{xy},Q_z) intensity maps and I(Q_{xy}) Bragg peak profiles integrated over all Q_z values for MRS and MDS membranes doped in TBBPA. a) MRS, X(TBBPA) = 0.1, b) MRS, X(TBBPA) = 0.2, c) MDS, X(TBBPA) = 0.1, d) MDS, X(TBBPA) = 0.2. The measurements were performed at π = 20 mN/m, solid lines in Bragg peak profiles are Lorentz curves fitted to the experimental data.

Table S1. Structural parameters extracted from the GIXD data for the TBBPA-doped membranes.

System	Q_{xy}, Q_z (Å^{-1}, Å^{-1})	a, b, γ (Å, Å, deg)	A_{xy} (Å^2)	τ (deg)	L_{xy} (Å)	I (a.u.)
MRS	<-1,1> 1.452; 0.46<br><0,2> 1.484; 0	4.979; 8.400; 90	20.91	20.0	126±3<br>395±8	860
MRS, X(TBBPA)=01	<0,1> 1.454; 0.47<br><1,0> 1.476; 0.38<br><-1,1> 1.496; 0.02	4.849; 4.922; 118.6	20.95	18.9	184±6<br>205±15<br>461±12	911
MRS, X(TBBPA)=02	<0,1> 1.458; 0.47<br><1,0> 1.479; 0.41<br><-1,1> 1.497; 0.02	4.844; 4.914; 118.7	20.90	19.3	168±5<br>240±21<br>461±10	802
MDS	<-1,1> 1.484; 0.33<br><0,2> 1.504; 0	4.911; 8.355; 90	20.51	14.5	163±4<br>395±7	1434
MDS, X(TBBPA)=01	<0,1> 1.483; 0.35<br><0,2> 1.503; 0	4.915; 8.361; 90	20.55	15.3	178±5<br>225±5	1053
MDS, X(TBBPA)=02	<0,1> 1.479; 0.37<br><0,2> 1.502; 0	4.931; 8.366; 90	20.63	16.2	173±5<br>395±8	1285

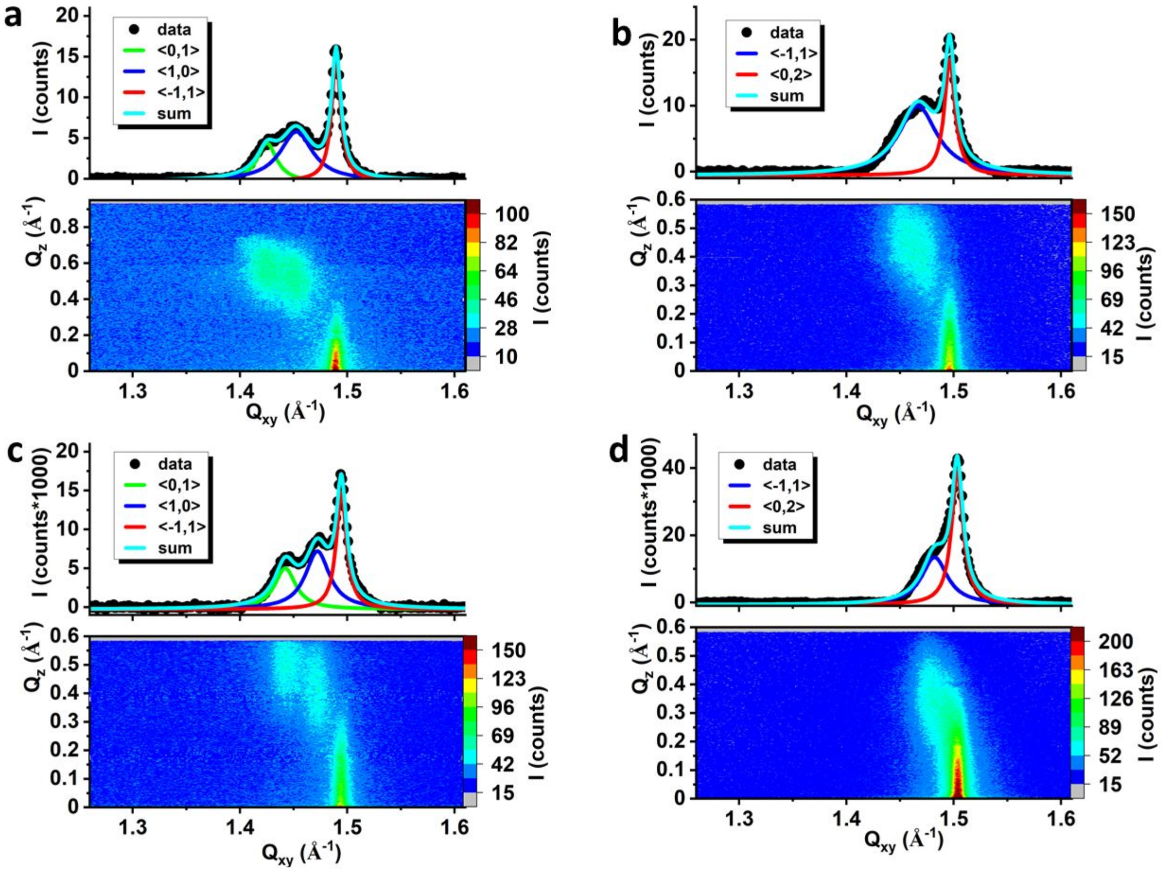

Figure S15. GIXD results: I(Q_{xy},Q_z) intensity maps and I(Q_{xy}) Bragg peak profiles integrated over all Q_z values for MRS and MDS membranes doped in HBCD, X(HBCD) = 0.1 in all the experiments. a) MRS, π = 10 mN/m, b) MRS, π = 20 mN/m, c) MDS, π = 10 mN/m, d) MDS, π = 20 mN/m. Solid lines in Bragg peak profiles are Lorentz curves fitted to the experimental data.

Table S2 Structural parameters extracted from the GIXD data for the HBCD-doped membranes.

**System**	**Q_{xy}, Q_z (Å^{-1}, Å^{-1})**	**a, b, γ (Å, Å, deg)**	**A_{xy} (Å^2)**	**τ (deg)**	**L_{xy} (Å)**	**I (a.u.)**
MRS,<br>π=10 mN/m	<0,1> 1.433; 0.58<br><1,0> 1.464; 0.49<br><-1,1> 1.498; 0.02	4.849; 4.954; 117.7	21.26	23.4	197±21<br>162±9<br>595±32	363
MRS,<br>X(HBCD)=0.1,<br>π=10 mN/m	<0,1> 1.425; 0.58<br><1,0> 1.453; 0.50<br><-1,1> 1.490; 0.02	4.882; 4.978; 117.7	21.53	23.7	251±22<br>149±8<br>553±9	620
MRS,<br>π=20 mN/m	<-1,1> 1.452; 0.46<br><0,2> 1.484; 0	4.979; 8.400; 90	41.82<br>(20.91)	20.0	126±3<br>395±8	860
MRS,<br>X(HBCD)=0.1,<br>π=20 mN/m	<-1,1> 1.466; 0.47<br><0,2> 1.496; 0	4.983; 8.400; 90	41.86<br>(20,93_	20.4	126±3<br>461±13	861
MDS,<br>π=10 mN/m	<0,1> 1.453; 0.50<br><1,0> 1.479; 0.38<br><-1,1> 1.498; 0.02	4.837; 4.923; 118.6	20.92	19.7	251±9<br>197±7<br>425±10	658
MDS,<br>X(HBCD)=0.1,<br>π=10 mN/m	<0,1> 1.442; 0,51<br><1,0> 1.472; 0,41<br><-1,1> 1.495; 0,02	4.834; 4.943; 118.19	21.10	20.5	230±9<br>205±2<br>503±9	698
MDS,	<-1,1> 1.484; 0.33	4.911; 8.355; 90	20.51	14.5	163±4	1434

π=20 mN/m	<0,2> 1.504; 0				395±7	
MDS,<br>X(HBCD)=0.1,<br>π=20 mN/m	<-1,1> 1.482; 0.37<br><0,2> 1.503; 0	4.919; 8.361; 90	20.56	16.2	191±7<br>425±9	1328

Table S3 Structural parameters extracted from the GIXD data for the BTBPE-doped membranes.

System	Q_{xy}, Q_z (Å^{-1}, Å^{-1})	a, b, γ (Å, Å, deg)	A_{xy} (Å^2)	τ (deg)	L_{xy} (Å^2)	I (a.u.)
MRS,<br>π=10 mN/m	<0,1> 1.433; 0.58<br><1,0> 1.464; 0.49<br><-1,1> 1.498; 0.02	4.849; 4.954; 117.7	21.26	23.4	197±21<br>162±9<br>595±32	363
MRS,<br>X(BTBPE)=0.1<br>π=10 mN/m	<-1,1> 1.467; 0.48<br><0,2> 1,502; 0	4.987; 8.361; 90	41.70<br>(21,35)	20.9	108±4<br>503±18	385
MDS,<br>π=10 mN/m	<0,1> 1.453; 0.50<br><1,0> 1.479; 0.38<br><-1,1> 1.498; 0.02	4.837; 4.923; 118.6	20.92	19.7	251±9<br>197±7<br>425±10	658
MDS,<br>π=20 mN/m	<-1,1> 1.484; 0.33<br><0,2> 1.504; 0	4.911; 8.355; 90	41.03<br>(20.51)	14.5	163±4<br>395±7	1434
MDS,<br>X(BTBPE)=0.1,<br>π=10 mN/m	<0,1> 1.450; 0.48<br><1,0> 1.476; 0.41<br><-1,1> 1.496; 0.02	4.844; 4.931; 118.50	20.99	19.8	240±10<br>191±13<br>461±15	637
MDS,<br>X(BTBPE)=0.1,<br>π=20 mN/m	<-1,1> 1.494; 0.34<br><0,2> 1.513; 0	4.877; 8.306; 90	40.51<br>(20,25)	14.8	191±6<br>369±10	1100
MDS,<br>X(BTBPE)=0.1,<br>π=30 mN/m	<-1,1> 1.493, 0	4.859, 120	20.45	0	425+-4	1284
MDS,<br>X(BTBPE)=0.2<br>π=10 mN/m	<-1,1> 1.479; 0.46<br><0,2> 1.506; 0	4.936; 8.344; 90	41.19	19.9	154±9<br>253±11	460

5. Selected BAM images for the BTBPE-doped POPE monolayer

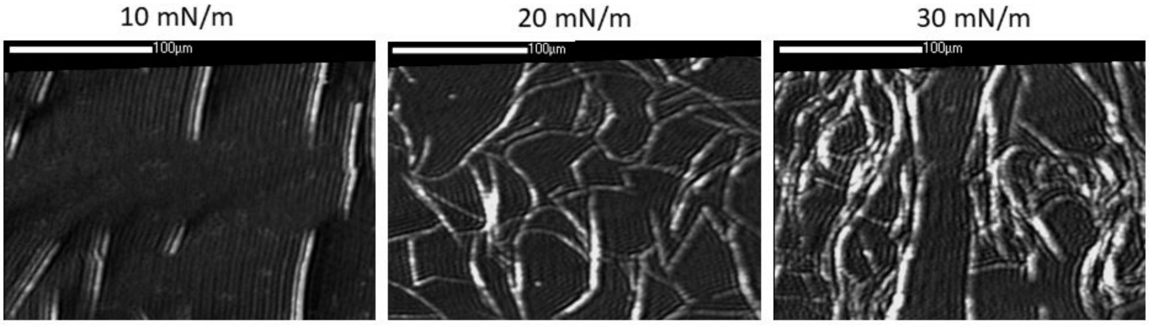

Figure S16. Selected BAM images for the BTBPE-doped POPE monolayer. X(BTBPE) = 0.1. The scale bar is 100 μm.
